# UV-Induced DNA Repair Mechanisms and Their Effects on Mutagenesis and Culturability in *Escherichia coli*

**DOI:** 10.1101/2024.11.14.623584

**Published:** 2024-11-14

**Authors:** Sreyashi Ghosh, Mehmet A. Orman

**Affiliations:** William A. Brookshire Department of Chemical and Biomolecular Engineering, University of Houston, Houston, TX, USA

## Abstract

Mutagenic processes drive evolutionary progress, with ultraviolet (UV) radiation significantly affecting evolution. Despite extensive research on SOS response-mediated mutagenesis, UV-induced repair mechanisms remain complex, and their effects on cell survival and mutagenesis are not fully understood. We previously observed a near-perfect correlation between RecA-mediated SOS response and mutation levels in *Escherichia coli* following UV treatment. However, prolonged UV exposure caused transient non-culturability and impaired SOS-mediated mutagenesis. Using fluorescent reporters, flow cytometry, promoter-reporter assays, single-gene deletions, knockouts, and clonogenic assays, we found that excessive UV exposure disrupts cellular translation, reducing SOS gene expression, albeit with minimal impact on membrane permeability or reactive oxygen species levels. While our findings underline the abundance of repair mechanisms in *E. coli* cells, enabling them to compensate when specific genes are disrupted, they also highlighted the differential impacts of gene deletions on mutagenesis versus culturability, leading to three major outcomes: (i) Disruption of proteins involved in DNA polymerase for translesion synthesis (UmuC and UmuD) or Holliday junction resolution (RuvC) results in significantly decreased mutagenesis levels while maintaining a transient non-culturability pattern after UV exposure. (ii) Disruption of proteins involved in homologous recombination (RecA and RecB) and nucleotide excision repair (UvrA) leads to both significantly reduced mutagenesis and a more severe transient non-culturability pattern after UV exposure, making these cells more sensitive to UV. (iii) Disruption of DNA Helicase II (UvrD), which functions in mismatch repair, does not affect mutagenesis levels from UV radiation but results in a very pronounced transient non-culturability pattern following UV exposure. Overall, our results further advance our understanding of bacterial adaptation mechanisms and the role of DNA repair pathways in shaping mutagenesis.

## Author Summary

Ultraviolet (UV) radiation has been a significant force in driving genetic variation and adaptation throughout billions of years of evolution. By directly damaging DNA and triggering repair mechanisms, UV radiation is a powerful tool for studying mutagenesis. This study aims to provide new insights into the complex processes behind bacterial mutagenesis, a critical topic in microbiology and public health. Although the connection between the SOS response and DNA repair in UV-treated cells is well known, two key phenomena — UV-induced bacterial cell dormancy and mutagenesis — remain poorly understood. Our findings reveal a highly unusual, SOS response-dependent transient non-culturability in *Escherichia coli* cells following prolonged UV exposure. However, the downstream mechanisms of this phenomenon and its links to mutagenesis remain unclear. This study seeks to thoroughly investigate these phenomena, offering new insights into their underlying molecular processes.

## Introduction

Mutagenic processes are key to evolutionary progress ^1–3^, as they generate genetic diversity. This is essential for organisms to adapt to and endure in dynamic settings that are continuously changing. Understanding the mechanisms underlying mutagenesis helps us better comprehend the evolutionary forces shaping organisms ^4,5^. This knowledge also aids in developing more potent and effective medical treatments to combat many diseases, given that pathogenic microorganisms and tumorigenic cells develop resistance through mutagenesis^6–9^.

Ultraviolet (UV) radiation has continually shaped the trajectory of evolution over billions of years, from contributing to the origins of life to driving genetic variation and adaptation^10,11^. This is because UV radiation directly damages DNA and induces certain repair mechanisms that are highly mutagenic^12–15^. The UV light spectrum, ranging from 100 to 400 nanometers (nm), can be categorized into four groups: UV-A (315–400 nm), UV-B (280–315 nm), UV-C (100–280 nm), and vacuum-UV (100–200 nm)^16^. UV-C is more readily absorbed by nucleic acids compared to UV-B and UV-A^17^. When bacteria are exposed to UV radiation, ^6,7^ including UV-C, UV-B, and UV-A, it triggers the formation of pyrimidine dimers and other DNA lesions that disrupt the normal structure and function of DNA molecules^18,19^. These UV lesions are removed by various effective bacterial systems, such as the photoreactivation repair system, (i.e. dimer monomerization under visible light by photolyase enzymes^20^), base excision repair (BER) (removal of cyclobutane-thymine dimers using DNA glycosylases and AP endonucleases)^21^, and the primary pathway for removal of bulky DNA lesions, i.e. the nucleotide excision repair (NER) (for rapid repair of photo-lesions using the *uvr* genes, *polA* and ligase enzyme) ^22^. However, an excessive amount of dimers that cannot be removed by the repair systems can accumulate and interfere with the cellular replication process, resulting in the formation of “secondary” lesions in the form of ssDNA fragments^23^. Therefore, to deal with this severe DNA damage, a post-replication damage repair mechanism known as the bacterial SOS response pathway is induced, preventing premature cell division ^12,24,25^ and providing cells with sufficient time to repair the damaged DNA^14,26–28^. This SOS response in bacteria is controlled by the multifunctional RecA protein, a key component that cleaves the transcriptional repressor LexA^29–31^. This cleavage initiates the expression of more than 40 SOS genes. RecA, involved in the recombination repair of ssDNA gaps^32^, helps provide an error-free damage removal route. However, excessive DNA damage leads to the induction of the mutagenic phase of the SOS response, mediated by bacterial DNA polymerase enzymes, including Pol V ^33,34^, which is encoded by the *umuD* and *umuC* genes ^35^. Pol V can bypass template lesions during DNA replication through a process known as translesion DNA synthesis (TLS) ^36^. Two additional TLS DNA polymerases, Pol II (encoded by *polB*) and Pol IV (encoded by *dinB*), facilitate replication past blocking lesions, albeit at the cost of introducing mutations. Another DNA damage repair strategy is the mismatch repair (MMR) pathway ^37^, which recognizes base-base mismatches and insertion/deletion loops, including those introduced by DNA polymerases and as part of TLS during the SOS response. However, with increased mutagenic stress, the efficiency of MMR decreases, as observed in the case of fluoroquinolone treatment ^38^.

UV radiation can serve as a valuable experimental tool for investigating mutagenesis because it induces a wide range of mutations without an apparent sequence preference^39,40^. Additionally, UV radiation is of great interest to scientists for its applications in biotechnology, particularly in directed evolutionary strategies to engineer proteins or organisms^41,42^. Moreover, there is growing interest in using UV radiation as a disinfectant, including for sterilizing air, equipment, and surfaces; reducing the transmission of airborne diseases; and even treating wound infections^43,44^. Despite their increased applications in clinical settings, we believe that two phenomena— UV-induced bacterial cell dormancy and mutagenesis— need to be fully understood. Bacterial cells are highly heterogeneous, and they can enter a growth-arrested state through stochastic mechanisms or environmental factors^45^, including UV treatment, which can make these cells highly tolerant. Also, the SOS-response-mediated cell dormancy not only promotes cell survival but also sustains mutagenic processes, as dormant cells may still harbor DNA damage^46,47^. Additionally, UV radiation itself is highly mutagenic; it can accelerate the emergence of more resilient mutants, which could pose a serious threat to public health.

UV-induced repair mechanisms are highly complex as UV can damage many cellular components, including DNA, RNA, lipids, and proteins^48,49^. Although the role of the SOS response in mutagenesis has been extensively studied (refer to the following review articles^12,26,28^ for more details), our understanding of the downstream repair mechanisms and their contributions to cell survival, culturability, and mutagenesis remains limited. In our recent study^50^, we observed a significant correlation between the RecA-mediated SOS response and mutation levels. Additionally, prolonged UV exposure was found to impair SOS-mediated mutagenesis and induce a transient non-culturability state ^50^, which may represent a potential survival strategy for cells to evade UV-induced mutagenesis. However, the underlying mechanisms remain unclear. Our current study aims to investigate these complex mechanisms further, providing new insights into the molecular processes underlying mutagenesis and dormancy.

## Results and Discussion

### The transient unculturability is largely driven by the global regulator RecA, crucial for the SOS response and recovery

**Fig. 1:**
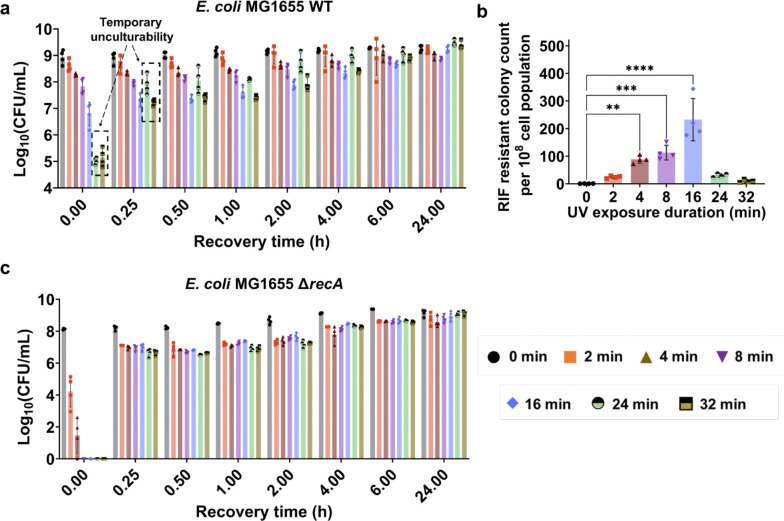
Excessive UV exposure resulted in transient loss of culturability and decreased mutagenesis. (a) Exponential-phase *E. coli* MG1655 WT cells were exposed to UV-B light for 0, 2, 4, 8, 16, 24, and 32 minutes, followed by a 24-h recovery period. At specific time points during recovery (t = 0 h, 0.25 h, 0.5 h, 1 h, 2 h, 4 h, 6 h, and 24 h), cells were collected and plated to determine colony-forming units (CFU). (b) Levels of UV-induced rifampicin (RIF) resistance mutations were measured by counting RIF-resistant colonies (per 10^8^ cells) in the WT cultures after recovery for the indicated UV exposure times. (c) The temporal CFU profiles of *E. coli* MG1655 Δ*recA* cells were monitored during recovery following UV treatment. n=4. Statistical analysis was performed using one-way ANOVA with Dunnett’s post-test, where *P < 0.05, **P < 0.001, ***P < 0.01, ****P < 0.0001. Data corresponding to each time point represent mean value ± standard deviation.

To study UV-mediated mutagenesis and the SOS response, we used our previously established methodology^50^. We cultured *E. coli* MG1655 wild-type cells in 2 mL Lysogeny Broth (LB) medium in test tubes until they reached the mid-exponential phase with an optical density (OD_600_) of ~0.5. Subsequently, the cells were transferred to petri dishes, forming a thin film of cultures that increased the surface area for UV exposure. This film was then subjected to UV-B light (302 nm thin-line transilluminator, UV intensity ~2000 μW/cm^2^, UVP ChemStudio, Analytik Jena) for various durations: 2, 4, 8, 16, 24, and 32 minutes (min). Following exposure, the cells were promptly transferred back to test tubes and cultured in a shaker for 24-hour (h) recovery (refer to the Materials and Methods section for details). Cultures that did not receive UV-B treatment served as controls. During recovery, colony-forming units (CFU) were quantified at the indicated time points for each condition, as shown in Fig. 1a. At the beginning of recovery (t~0), CFU levels decreased in cultures exposed to longer UV-B treatments. A 4–8 min exposure caused a 10-fold reduction, while 16 min led to a ~100-fold drop compared to the control. Exposure times of 24 and 32 min resulted in a ~10,000-fold reduction (Fig. 1a). After 15 minutes, CFU levels notably increased in cultures with 24 and 32 min of UV-B exposure, likely due to mechanisms making cells temporarily unculturable. This drastic increase in CFU levels is not due to cell division, given that the *E. coli* doubling time is about 20–25 minutes, which is too short for significant growth within that period. By the 24-hour mark, all conditions showed similar CFU levels (Fig. 1a).

To measure mutagenesis, we quantified rifampicin (RIF)-resistant colonies after 24 hours of recovery. Cultures were plated on LB agar with 500 μg/ml RIF, and colony counts were reported per 10^8^ cells (Fig. 1b). Although this method does not directly measure mutation levels, it is widely used ^40,51–53^ due to the strong correlation between mutagenesis and resistant colony formation ^54,55^. We chose a 24-hour recovery period to ensure accurate colony counts, consistent with previous studies^51,56,57^, as mutation levels generally plateau by this time^50^. Our results showed that mutagenesis increased with UV-B exposure, peaking at 16 minutes, but longer exposures (24–32 min) significantly reduced RIF-resistant colonies (Fig. 1b). This pattern, consistent with our previous findings^50^, suggests a link between UV-B exposure, mutagenesis, and temporary unculturability.

In our previous study, we found that excessive UV-B treatment (24 and 32 minutes) impaired the SOS response, including the expression level of the RecA protein^50^. Therefore, the observed transient unculturability is likely more complex and may involve various mechanisms, given that the SOS response is intricate, involving multiple proteins, with RecA serving as the global regulator. We hypothesized that if the observed transient unculturability is linked to the impaired SOS response, then genetic perturbation of this response through *recA* deletion would further decrease cell culturability. Our results verified that the knockout strain of *recA* exhibited a drastic reduction in culturability immediately after UV treatment across all conditions (Fig. 1c). Initially, CFU levels in the Δ*recA* strain were below the limit of detection (1 CFU) for most UV treatment conditions; however, this was transient, as we observed a rapid increase in CFU levels (from 1 to 10^7^) within 15 minutes of recovery (Fig. 1c). This finding is highly interesting and, to our knowledge, has not been previously reported. As we have already shown the deletion *recA* eliminates the mutagenesis following UV treatment^50^, the impairment of the RecA-mediated SOS response during prolonged UV exposures^50^ should significantly affect downstream processes. However, it remains unclear how these downstream processes impact cell culturability and mutagenesis; thus, we aim to investigate these aspects in the subsequent sections of our study.

### Genes significantly expressed during the SOS response have a moderate or negligible effect on cell culturability and mutagenesis

To identify the downstream mechanisms, we screened a subset of the *E. coli* promoter library containing 1900 promoter reporters. In this library, the promoters of *E. coli* genes are fused to a fast-folding green fluorescent protein (GFP) gene in low-copy-number plasmids, allowing accurate and reproducible measurement of gene expression^58^. We focused on approximately 40 reporters containing promoters that regulate the key SOS response and associated genes. These promoters are under the control of the RecA-LexA regulatory system, chosen based on a comprehensive analysis of *E. coli* databases^59^. In our experiments, *E. coli* MG1655 cells containing the selected promoters were cultured individually until the mid-exponential phase in 96-well plates. Subsequently, they were exposed to UV-B radiation for 16 min. This duration was chosen as it induces maximum SOS upregulation and mutagenesis without drastically impacting cell culturability (Fig. 1a,b). Also, 16-min UV treatments were used as positive controls for the subsequent sections. GFP levels were measured after 24 h of recovery, allowing adequate time for UV-induced promoters to express GFP. High cell densities ensured reliable GFP measurements, given that we used a plate reader in this screening assay.

**Fig. 2:**
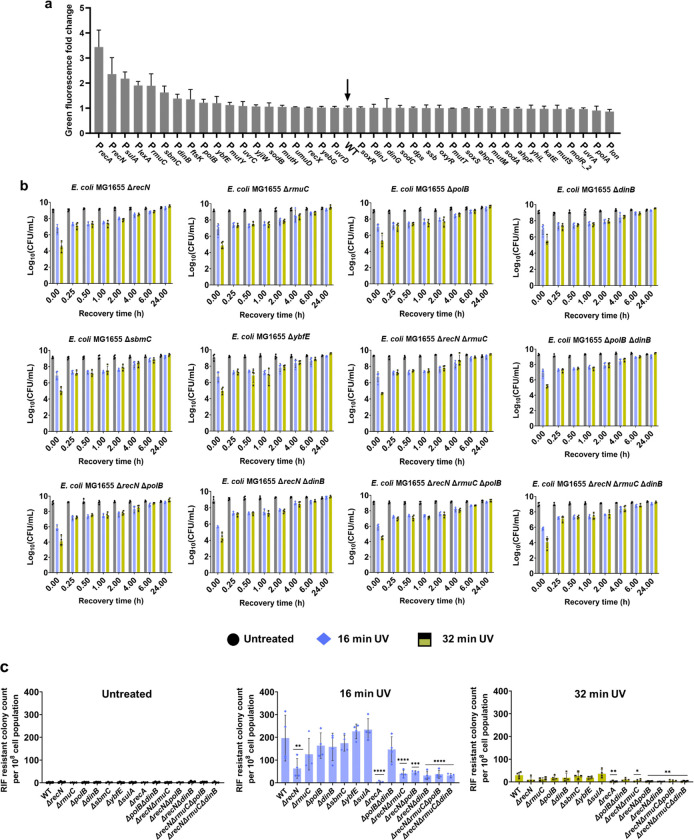
UV-induced upregulation of SOS genes and its impact on mutagenesis and cell culturability. (a) A library of *E. coli* MG1655 strains with promoter reporters for SOS response genes was UV-treated during the mid-exponential phase and allowed to recover for 24 h. GFP levels were measured after recovery and normalized to untreated controls. WT cells without any promoter reporters were used as background controls. (b) Genes upregulated following UV exposure were individually or combinatorially deleted in *E. coli* MG1655, and a mutagenesis assay was performed. Mid-exponential phase cells were exposed to UV for 0, 16, and 32 min and recovered for 24 h. At specified time points during recovery (t = 0 h, 0.25 h, 0.5 h, 1 h, 2 h, 4 h, 6 h, and 24 h), cells were collected and plated to determine CFU. (c) RIF-resistant cells were quantified by plating samples on RIF-agar plates after 24 h of recovery, with results reported as RIF-resistant colony counts per 10^8 cells for different UV exposure durations. n=4. Statistical analysis was performed using one-way ANOVA with Dunnett’s post-test, where *P < 0.05, **P < 0.001, ***P < 0.01, ****P < 0.0001. Data corresponding to each time point represents mean value ± standard deviation.

Our specific focus was on genes showing significant upregulation following UV treatment (Fig. 2a), as they likely play pivotal roles in the downstream mechanisms of RecA-mediated SOS response. Several promoters showed drastic upregulation, including P_*recA*_ and P_*lexA*_ (promoters of genes encoding SOS response global regulators), P_*recN*_ and P_*rmuC*_ (promoters of genes involved in DNA recombinational repair), P_*sulA*_ (promoter of a gene encoding cell division inhibitor), P_*polB*_ and P_*dinB*_ (promoters of genes encoding DNA polymerase enzymes), P_*ftsK*_ (promoter of a gene encoding essential cell division protein), P_*sbmC*_ (promoters of a gene encoding inhibitor of DNA gyrase-mediated DNA supercoiling), and P_*ybfE*_ (promoter of a *lexA-*regulated gene whose function is not well characterized)^60^ (Fig.2a). To determine whether the genes associated with the identified promoters are involved in SOS response-mediated culturability and mutagenesis, we generated single deletions in *E. coli* MG1655 for these genes (except for *lexA* and *ftsK*, as they are essential and could not be deleted^61–63^). The knockout strains were exposed to UV-B for 16 min or 32 min or untreated, and their CFU profiles during recovery, as well as their RIF-resistant colony levels, were similarly measured. For all knockout strains, the temporal CFU profiles remained the same as that of the wild-type, showing a transient non-culturability during the first 15 min of recovery after a high UV-B exposure duration of 32 min (Fig. 2b). As expected, the deletion of *recA* completely eliminated mutagenesis (Fig. 2c). Although 32 min of UV-B exposure significantly reduced mutagenesis in all strains, including wild type, the two DNA recombination gene knockouts, Δ*recN* and Δ*rmuC*, showed a 3-fold and 1.5-fold decrease in RIF-resistant mutant levels, respectively, compared to WT cells following 16 min of UV-B treatment (Fig. 2c). Surprisingly, other single deletion strains, including those for the genes encoding DNA polymerase II and IV, *dinB* and *polB*, did not show significant changes (Fig. 2c).

Overall, our results showed that single deletions of the downstream genes of RecA did not drastically impact cellular culturability and mutagenesis, except for RecN and RmuC deletions, which slightly reduced mutagenesis. RecN is involved in the recombinational repair of DNA double-strand breaks, and its mutation makes cells sensitive to mitomycin C and ionizing radiation^64–66^. Although RmuC is thought to be an inner membrane protein with a nuclease domain^67,68^, its function is not well understood, and our data indicate it only moderately impacts UV-B-mediated mutagenesis. It is quite surprising that the deletion of DNA polymerase II and IV, two key proteins involved in indirect mutagenesis^69^, did not impact UV-B-induced mutagenesis. As mutagenesis is a multifaceted process regulated by multiple mechanisms, testing multi-deletion strains might be necessary to show the complex interplay between these mechanisms. Furthermore, deleting a single polymerase enzyme may have minimal impact due to the abundance of other polymerase enzymes. We first generated Δ*recN*Δ*rmuC* and showed that the double deletion resulted in a cumulative decrease (~5 fold) in RIF-resistant colony levels compared to the wild type following 16 min of UV-B treatment (Fig. 2c). To assess the impact of DNA polymerase II and IV, we generated a Δ*polB*Δ*dinB* mutant strain, which did not affect RIF-resistant colony levels (Fig. 2c). Additionally, we generated Δ*recN*Δ*polB* and Δ*recN*Δ*dinB* double knockout strains and demonstrated that they exhibited approximately a 5-fold reduction in RIF-resistant colony levels compared to wild-type cells (Fig. 2c). Deletion of DNA polymerase genes *polB* and *dinB* from Δ*recN*Δ*rmuC* individually did not show significant changes compared to the Δ*recN*Δ*rmuC* strain in RIF-resistant mutant levels (Fig. 2c), suggesting that the observed reduction in RIF-resistant colonies in multi-deletion strains is primarily due to the effects of *recN* and/or *rmuC*.

### Screening the knockout strains reveals the redundancy of repair mechanisms in UV-induced mutagenesis and culturability

**Fig. 3:**
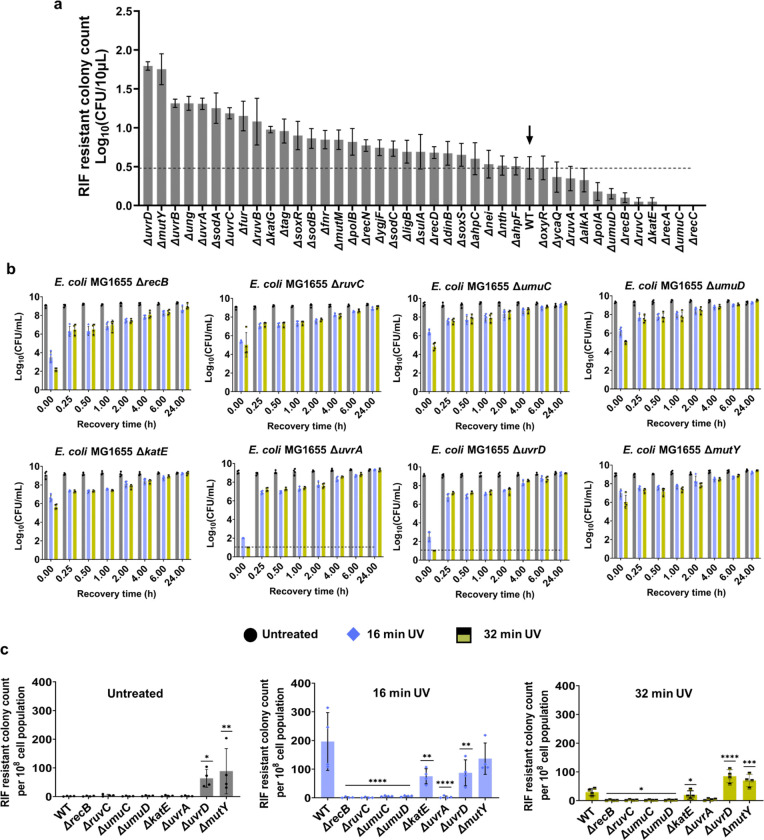
Knockout library screening to elucidate their effects on mutagenesis and cell culturability. (a) The *E. coli* BW25113 Keio knockout collection was screened following 16 min of UV treatment. RIF-resistant cells were quantified by spotting 10 μL samples from each well onto RIF-agar plates after a 24-hour recovery period. (b) Selected genes were deleted in *E. coli* MG1655 and the mutagenesis assay was performed. Mid-exponential phase cells were exposed to UV for 0, 16 and 32 min and recovered for 24 h. At indicated time points (t = 0 h, 0.25 h, 0.5 h, 1 h, 2 h, 4 h, 6 h and 24 h) during recovery, cells were collected and plated to enumerate CFU. (c) RIF-resistant cells were quantified by plating the samples on RIF-agar plates after 24 h of recovery. The values were reported as RIF-resistant colony count per 10^8^ cell population for different UV exposure durations. n=4. Statistical analysis was performed using one-way ANOVA with Dunnett’s post-test, where *P < 0.05, **P < 0.001, ***P < 0.01. Data corresponding to each time point represents mean value ± standard deviation.

Although our initial screening yielded intriguing results, the critical downstream mechanisms underlying our observed phenomenon still remain elusive. Thus, we conducted a second screening using the *E. coli* BW25113 Keio knockout collection to assess the impact of UV on mutagenesis and culturability of the knockout strains. This screening focused on all potential repair mechanisms involved^12^, particularly those (i) that are not regulated by RecA and (ii) whose expression was not significantly upregulated in our initial screening (Fig. 2a). For this screening, the knockout strains in 96-well plates were treated with UV-B for 16 min during mid-exponential-phase growth, and RIF-resistant colonies were quantified after a 24-hour recovery period. We identified several genes whose deletion resulted in a significant reduction in RIF-resistant colony formation (Fig. 3a), including *recB*, a component of Exonuclease V or the RecBCD complex that promotes homologous recombination in the repair of double-strand DNA breaks^70,71^; *umuC* and *umuD*, encoding error-prone DNA polymerase V^72^; *ruvC*, encoding an endonuclease that binds to and cleaves Holliday junctions^73^; and *katE*, encoding *E. coli* catalase enzyme^74^. Additionally, we observed several genes that showed an increase in mutagenesis, such as *uvrD* and *uvrA*, which are involved in the nucleotide excision repair pathway^75,76^, and *mutY*, involved in the base excision repair pathway^77^ (Fig. 3a).

To better understand how these genes affect mutagenesis and culturability, and to see if similar trends occur in other *E. coli* strains, we deleted these genes in *E. coli* MG1655 and analyzed the knockout strains after 16 and 32 min of UV-B exposure. Deletion of *recB*, *ruvC*, *umuC*, and *umuD* significantly reduced mutagenesis (Fig. 3c), consistent with our screening data. The absence of UmuC and UmuD proteins, specialized for translesion synthesis, was shown to sensitize *E. coli* cells to UV^78,79^, aligning with our findings. While Δ*uvrA* in the *E. coli* BW25113 strain from screening showed higher mutant levels compared to wild-type cells, its deletion in the MG1655 background resulted in a significant reduction in mutant formation (Fig. 3c), suggesting that the role of certain genes in mutagenesis can vary depending on the genetic background of the *E. coli* strain. Also, our results indicate that KatE, UvrD, and MutY may not be directly involved in the mutagenic response to UV radiation under the conditions tested here, as their deletions did not clearly impact UV-B-induced mutagenesis in *E. coli* MG1655 (Fig. 3c). Interestingly, Δ*uvrD* and Δ*mutY* strains exhibited increased RIF-resistant colonies in cultures not treated with UV-B (Fig. 3c). UvrD is involved in various DNA repair pathways, including NER and MMR processes^37,80,81^. MutY is a glycosylase enzyme that corrects adenine mismatches resulting from DNA replication errors, primarily in the BER pathway^77^. While the specific roles of UvrD and MutY in UV-B-induced mutagenesis are not evident in this study, the increased RIF-resistant colonies in untreated cultures might highlight their potential role in maintaining genomic stability under normal growth conditions, possibly by mitigating spontaneous mutations.

The temporal CFU profiles revealed interesting trends as well. Δ*ruvC*, Δ*umuC*, and Δ*umuD* strains showed no significant change in transient non-culturability immediately after UV-B treatment compared to the wild type (Fig. 3b). However, Δ*recB* exhibited a notable decrease in CFU levels, while Δ*uvrA* and Δ*uvrD* mutants showed even more drastic reductions following UV-B treatment compared to the wild type (Fig. 3c). This reduction in CFU levels was transient, as their levels increased approximately 10^7^ -fold within 15 min of recovery. UvrA and UvrD together form key components of the NER pathway^80^. UvrA forms a complex with UvrB to detect DNA lesions, while UvrD functions as a helicase to unwind and remove damaged DNA segments^81,82^. As both UvrA and UvrD are regulated by the RecA protein^28^, it is not surprising that the deletion of *recA* resulted in drastic reductions in CFU levels following UV treatment (Fig. 1c). Since RecA is essential for inducing the SOS response, its absence means that many DNA repair genes, including *uvrA* and *uvrD*, are not upregulated in response to UV-induced DNA damage.

### Excessive UV exposure impairs cellular translation processes

Although our results highlight specific SOS proteins involved in mutagenesis and cell culturability, it remains unclear why excessive UV exposure impairs *recA* expression and, consequently, the SOS response. This impairment could be due to the activation of cellular mechanisms, such as growth arrest proteins, which may help limit excessive DNA damage and mutagenesis. Additionally, the production of toxic molecules, such as reactive oxygen species, or the inhibition of certain cellular functions, like translation, may also contribute to this effect. These mechanisms—namely the involvement of growth arrest proteins, reactive oxygen species, or the inhibition of translation—will be thoroughly explored in the subsequent section of our study.

**Fig. 4:**
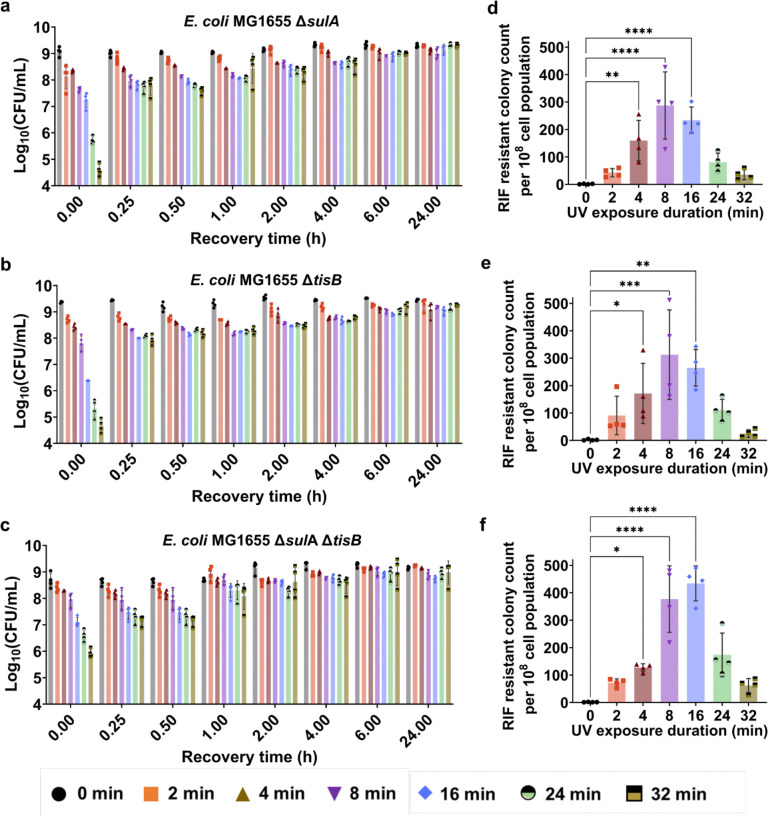
Perturbation of both SulA and TiB proteins moderately increased cell culturability during recovery. (a-c) Exponential-phase *E. coli* MG1655 Δ*sulA*, Δ*tisB*, and Δ*sulA*Δ*tisB* cells were exposed to UV-B light for 0, 2, 4, 8, 16, 24, and 32 minutes, followed by a 24-h recovery period. At specific time points during recovery, cells were collected and plated to determine their CFU levels. (d-f) Levels of UV-induced RIF resistance mutations were measured by counting RIF-resistant colonies (per 10^8^ cells) in the cultures of three knockout strains after recovery for the indicated UV exposure times. n=4. Statistical analysis was performed using one-way ANOVA with Dunnett’s post-test, where *P < 0.05, **P < 0.001, ***P < 0.01, ****P < 0.0001. Data corresponding to each time point represent mean value ± standard deviation.

Within the SOS response, two genes, *sulA* and *tisB*, play key roles in cell growth inhibition. The *sulA* gene in *E. coli* encodes a cell-division inhibitor that temporarily halts the cell cycle during the SOS response, preventing damaged DNA from prematurely segregating into daughter cells^83^. On the other hand, *tisB* encodes a toxin protein that reduces cellular ATP levels^46,84^. This protein may also induce reversible dormancy by suppressing cell metabolism. To investigate the individual and combined effects of these genes, we constructed knockout strains of *E. coli* MG1655 Δ*sulA*, Δ*tisB*, and Δ*sulA*Δ*tisB*. Similarly, these strains, at the mid-exponential phase, were exposed to varying durations of UV-B radiation followed by a 24-hour recovery period. *E. coli* MG1655 WT cells were used as the control group for comparison. Deleting *sulA* and *tisB* individually did not alter the trend of transient non-culturability observed in our experiments. Specifically, the CFU profiles initially showed a significant decrease after prolonged UV-B exposure of 24 and 32 min, followed by a sharp increase within the first 15 min of recovery, similar to the wild-type strain (Fig. 1a vs Fig. 4a–c). However, CFU levels of Δ*sulA*Δ*tisB* immediately after 24 and 32 min of UV-B exposure (corresponding to the t~0 point on the CFU plots) were nearly 10-fold higher compared to those of the wild-type or single knockout strains (Fig. 1a vs Fig. 4a–c). We observed approximately a 10-fold increase in CFU levels within the first 15 min of recovery in the double knockout strain, although this increase was not as pronounced as in the control or single knockout strains (Fig. 4a–c). While deleting both genes enhanced the culturability of cells following UV-B treatment, the phenomenon observed in the wild-type strain was not completely eliminated in the double knockout strain, highlighting the complexity of the underlying mechanisms. No distinct profiles of RIF-resistant mutants were observed after 24 hours of recovery in either single or double-knockout strains compared to the wild-type strain (Fig. 1b vs Fig. 4d–f), indicating that these genes are not directly involved in mutagenesis, although they may affect cellular culturability to some extent.

**Fig. 5:**
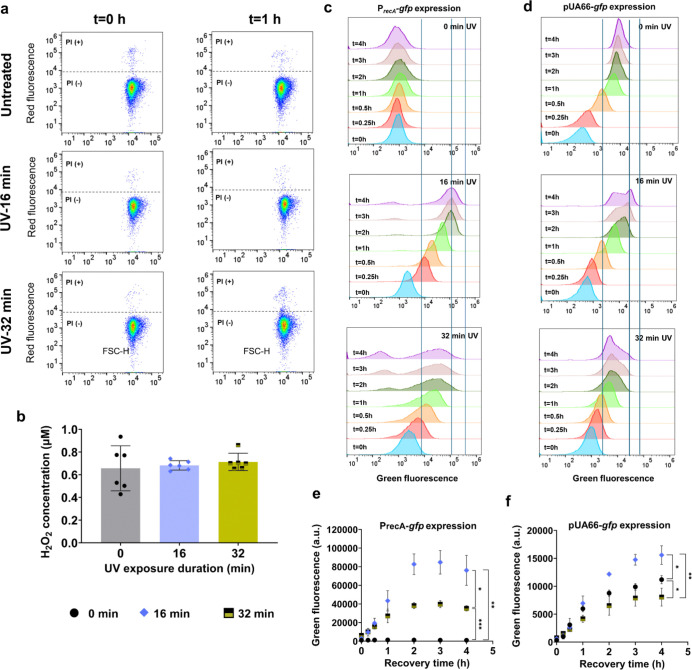
Excessive UV exposure did not compromise cell membrane integrity or increase hydrogen peroxide levels, but it did impair cellular translation. (a) Membrane integrity of UV-treated cells (0, 16, and 32 min of UV exposure) was assessed via flow cytometry with PI staining (see Supplementary Fig. S1 for controls). FSC-H: Forward light scatter. (b) H_2_O_2_ concentrations were measured using the Amplex Red Hydrogen Peroxide/Peroxidase Assay Kit. Reaction mixtures containing Amplex Red reagent, horseradish peroxidase, and UV-treated cells in sodium phosphate buffer (pH 7.4) were incubated for 30 minutes at room temperature, followed by fluorescence measurement with a microplate reader. H_2_O_2_ levels were calculated using a standard curve (Supplementary Fig. S2). (c) Expression of P_*recA*_-*gfp* in UV-treated *E. coli* MG1655 cells was analyzed via flow cytometry for the indicated time points during recovery (P_*recA*_ : the *recA* promoter). (d) IPTG-inducible GFP expression in UV-treated *E. coli* MG1655 pUA66-*gfp* cells was also assessed by flow cytometry for the indicated time points during recovery. (e) GFP expression profiles of UV-treated cells harboring the *recA* promoter and (f) IPTG-inducible GFP expression systems were evaluated using mean GFP values from flow cytometry data. Note that the vertical lines in the flow diagrams roughly represent the upper, lower, and peak fluorescence levels of the overall cell population exposed to the 16-min UV-B treatment (corresponding to the 4-h treatment time point). These lines were included to help readers more easily distinguish the outcomes of different experimental conditions. n ≥ 4. Statistical analysis was performed using one-way ANOVA with Dunnett’s post-test, where *P < 0.05, **P < 0.001, ***P < 0.01, ****P < 0.0001. Data corresponding to each time point represent mean value ± standard deviation.

UV radiation has the potential to damage cellular membranes and compromise essential cellular functions^15^. This damage can lead to increased membrane permeability, leakage of cellular contents, and, ultimately, cell death^85,86^. Additionally, prolonged exposure to UV radiation can trigger excessive production of reactive oxygen species (ROS) within cells^87–89^. ROS are highly reactive molecules that can cause oxidative damage to cellular components such as DNA, proteins, and lipids, impairing cellular functions. It is possible that the 24-minute and 32-minute UV-B treatments studied here may not be severe enough to kill the bacterial cells completely, but they are likely sufficient to hinder cell growth and mutagenic processes by disrupting vital metabolic processes. To characterize cellular membrane permeability, cells treated with UV-B were immediately collected and incubated with propidium iodide (PI), a fluorescent dye that enters cells with compromised membranes. Flow cytometry data indicated no significant increase in permeabilization following prolonged UV-B treatment (32 min) up to 1 hour of recovery (Fig. 5a), compared to both untreated cells and moderately UV-treated cells (16 min), which did not markedly reduce CFU levels. Furthermore, when UV-treated samples were subjected to Amplex Red reagent (see Materials and Methods for details) to detect hydrogen peroxide (H₂O₂), a primary reactive oxygen species, no significant changes were observed in the 32-minute UV-treated cells compared to untreated or 16-minute UV-treated cells (Fig. 5b), indicating H₂O₂ may not be a key player. In our previous study, we found that ROS significantly affects cell culturability only when their concentrations are much higher than physiological levels, and their impact on cell culturability was observed to be non-transient ^50^.

**Fig. 6:**
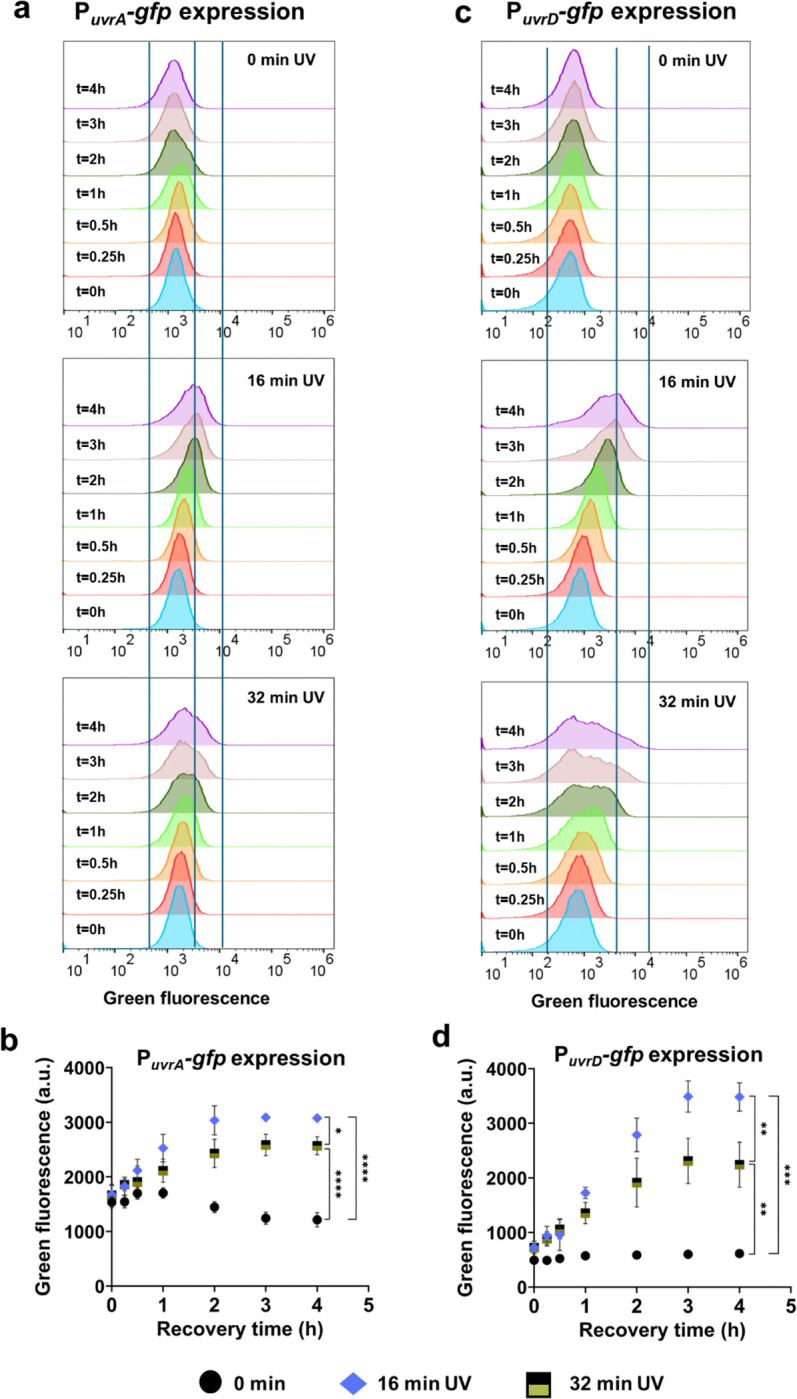
Flow cytometry analysis of nucleotide excision repair genes followed by UV-treatment (a, b) The GFP profiles of UV-treated cells harboring P_*uvrA*_-*gfp*, and (c, d) the GFP profiles of UV-treated cells harboring P_*uvrD*_-*gfp* were assessed using flow cytometry after UV exposure (for the indicated time points during recovery). Note that the vertical lines in the flow diagrams roughly represent the upper, lower, and peak fluorescence levels of the overall cell population exposed to the 16-min UV-B treatment (corresponding to the 4-h treatment time point). These lines were included to help readers more easily distinguish the outcomes of different experimental conditions. n=4. Statistical analysis was performed using two-way ANOVA, where *P < 0.05, **P < 0.001, ***P < 0.01, ****P < 0.0001. Data corresponding to each time point represent mean value ± standard deviation.

UV exposure is known to interfere with ribosomal function by inducing crosslinks in ribosomal RNAs^90^, which can inhibit overall translation processes and impair the expression of SOS response genes. To assess the effects of UV treatments on cellular translation, we analyzed GFP expression under the control of the *recA* promoter (P_*recA*_) at the single-cell level using *E. coli* MG1655 cells carrying the pUA66 P_*recA*_-*gfp* plasmids. The cells were subjected to UV-B treatments for 16 min and 32 min, and *recA* expression was measured using flow cytometry during the early recovery phase (Fig. 5c). UV-B treatment should activate promoters of SOS response genes and induce *recA* expression. As expected, the 16-min UV-B treatment resulted in significantly higher *recA* expression compared to untreated cells (Fig. 5c, e). However, the 32-min UV-B exposure resulted in more heterogeneous and lower *recA* expression compared to the 16-min UV-B exposure (Fig. 5c, e), potentially implying an impairment in the translational process. This difference between the 16- and 32-min treatments cannot be solely attributed to increased cell death following 32 min of UV-B exposure. Despite the observed transient culturability in the prolonged UV-exposure culture, CFU levels in both conditions are nearly similar after the brief transient period, around 15 minutes of recovery culture (see the 0.25-hour time point in Fig. 1a). To substantiate the hypothesis regarding the impairment of the translational process, we employed a GFP-expressing plasmid where the GFP gene is tightly regulated by an isopropyl β-D-1-thiogalactopyranoside (IPTG)-inducible T5 promoter and a strong LacI^q^ repressor^91^. Our rationale was that if excessive UV-B treatment indeed impairs translational processes, the levels of GFP expression in cells treated with 32 min of UV-B should be lower compared to those in the no-UV control or 16-minute UV-treated cells, despite the presence of the inducer. We supplemented cell cultures with 0.1 mM IPTG immediately before UV-B treatments. As anticipated, the 32-min UV-B treatment resulted in a lower level of GFP expression compared to the no-UV control or the 16-minute UV-B treatment (Fig. 5d, f). However, the 16-min UV-B treatment exhibited the highest GFP expression levels (Fig. 5d, f), suggesting that moderate UV treatment (16 min) enhances translational activity, whereas excessive UV-B treatment (32 min) slightly inhibits it. This moderate inhibitory effect of excessive UV-B treatment on cellular translation, including RecA expression, likely impacts overall cellular processes and compromises the SOS response. This was further validated when we measured the expression levels of *uvrD* and *uvrA*—two critical genes involved in mutagenesis and culturability. Their expression levels were slightly reduced under 32 minutes of UV treatment compared to 16 minutes (Fig. 6a–d).

Altogether, our findings suggest that while prolonged UV-B treatments may not directly impact membrane integrity or ROS levels, the observed unculturability of the cells likely results from a combined effect of multiple factors, including cell growth inhibitors, impaired translation processes, and downstream repair pathways. The impairment of cellular processes such as translation may hinder the SOS response, as evidenced by the decreased expression of RecA in cells treated with 32 min of UV-B (Fig. 5e). This interpretation is further supported by our findings with the Δ*recA* strain, which exhibited near-complete unculturability immediately after UV treatments. The immediate reduction in CFU levels in the knockout strains (Δ*recA*, Δ*uvrA* and Δ*uvrD*), as well as in the wild-type strain exposed to prolonged UV, highlights the importance of the SOS response in the initial phases of DNA damage repair. The transient nature of CFU reduction in all these mutants also suggests that *E. coli* possesses redundant or numerous repair mechanisms^12^ that can partially restore culturability even when certain DNA repair pathways are disrupted.

## Conclusions

In this study, we investigated the effects of UV-B treatments on *E. coli* mutagenesis and culturability and provided important insights into bacterial survival mechanisms under stress. Mutagenesis, measured by RIF-resistant colony formation, peaked at 16 min of UV-B exposure but decreased with longer exposures (24 and 32 min). The longer UV-B exposures also led to a significant decrease in CFU levels, but this phenomenon was observed to be a transient state, as we saw a drastic jump in CFU levels after a short recovery period. We also explored the role of key SOS response genes and demonstrated that knockout strains lacking *sulA* and *tisB* genes exhibited similar patterns of transient non-culturability as wild-type cells, although double knockouts exhibited higher initial CFU levels post-exposure, indicating a partial role in culturability. Further investigation revealed that excessive UV exposure did not significantly affect membrane permeability or reactive oxygen species (ROS) levels but moderately impaired translation processes, as evidenced by reduced expression of SOS response genes like *recA* or IPTG-inducible fluorescent proteins. This impaired translation likely contributed to the observed reduction in mutagenesis and transient non-culturability following prolonged UV exposure, given the significant impact of *recA* deletion on cell culturability and mutagenesis.

Although our screening of promoter reporters highlighted several genes significantly upregulated following UV treatment, the knockout strains of most of these genes generally had minimal impact on culturability or mutagenesis, except for Δ*recN* and Δ*rmuC*, which moderately reduced mutagenesis. In our second screening with the Keio knockout collection, we identified several critical genes, such as *recB, umuC, umuD*, and *ruvC*, whose deletions led to decreased mutagenesis, highlighting their crucial roles in facilitating error-prone repair mechanisms during UV-B stress. Furthermore, we observed differential impacts of gene deletions on mutagenesis versus culturability, providing insights into additional functions of these genes. Particularly in strains lacking *uvrA*, or *uvrD* showed a drastic but transient reduction in their culturability following UV treatment, highlighting their role in maintaining genomic stability. Our findings also underline the abundance of repair mechanisms in *E. coli*, demonstrating that multiple independent pathways can significantly impact cell mutagenesis and culturability. Overall, this study contributes to understanding bacterial responses to environmental stressors and the complex interactions of DNA repair pathways in maintaining genomic integrity, culturability, and survival.

## Materials and Methods

### Bacterial strains and plasmids

*Escherichia coli* K-12 MG1655 wild type (WT), and pUA66 plasmids with genes encoding a green fluorescent protein (*gfp*) under the control of promoter, P_*recA*_ were obtained from Dr. Mark P. Brynildsen at Princeton University. *E. coli* K-12 MG1655 Δ*sulA,* Δ*tisB,* Δ*recA* was constructed in our previous studies ^50,92,93^. The promoter strain collection of *E. coli* MG1655 in a 96-well plate format used for the screening assay was obtained from Horizon Discovery, Lafayette, CO, USA. The Keio knockout strain collection (a single-gene deletion library of E. coli K-12 BW25113) was obtained from Dharmacon Keio Collection (Dharmacon, Cat# OEC4988). The strains from both the collections used in this study are listed in Supplementary Table 1. The knockout *E. coli* MG1655 strains generated for this study are detailed in Supplementary Table 2. The method of Datsenko and Wanner^94^ was used to generate these strains and the oligonucleotides used to delete the genes are provided in Supplementary Table 3.

### Chemicals, media, and culture conditions

Unless otherwise specified, all chemicals were procured from Fisher Scientific (Atlanta, GA), VWR International (Pittsburgh, PA), or Sigma Aldrich (St. Louis, MO). PI staining kit was purchased from Promega Corporation (Madison, WI). *E. coli* cells were cultured in liquid Lysogeny-Broth (LB) medium. LB agar medium was utilized for enumerating colony-forming units (CFU) of *E. coli*. The liquid LB medium was prepared by dissolving 5 g yeast extract, 10 g tryptone, and 10 g sodium chloride in 1 L of deionized (DI) water. LB Agar media were prepared by dissolving pre-mixed 40 g LB agar in 1 L of DI water. Both solid and liquid media were subjected to autoclaving for sterilization.

Kanamycin (50 μg/mL) was included in the liquid LB media for plasmid selection and retention. IPTG at 0.1 mM was used to induce *gfp* expression. For PI staining, sterile 0.85% sodium chloride solution was used. When necessary, cells were washed with phosphate-buffered saline (PBS, 1X). Stock solutions for rifampicin (RIF; 50 mg/mL) was prepared by dissolving in DI water using 0.01 N sodium hydroxide. The IPTG stock solution (1 mM) was dissolved in DI water. All chemical solutions were sterilized using 0.2 μm VWR syringe filters.

To prepare RIF-agar plates, the stock RIF solution was added to autoclaved LB agar, resulting in a final plate concentration of 500 μg/mL RIF. Unless specified otherwise, overnight pre-cultures were generated in 14-mL Falcon test tubes containing 2 mL of liquid media. These pre-cultures were inoculated from a 25% glycerol cell stocks stored at −80 °C and cultivated for 24 h at 37 °C with shaking at 250 revolutions per min (rpm). Experimental cell cultures were prepared by diluting the overnight pre-cultures (1:100) into 2 mL of fresh LB medium in 14-mL Falcon test tubes. Bacterial cells in this study reached the mid-exponential phase (OD_600_ ~ 0.5) after around 3 h, attaining an average cell density of 7×10^8^ CFUs/ml. All treatments involving UV were administered at this stage.

### UV treatment and cell recovery

Overnight pre-cultures of *E. coli* MG1655 cells were diluted 100-fold in 2 mL fresh LB media in test tubes and grown at 37°C with shaking (250 rpm). Cell growth was monitored by measuring optical density at 600 nm wavelength (OD_600_) with a plate reader (Varioskan LUX Multimode Microplate Reader, Thermo Fisher, Waltham, MA, United States). When the cell density reached OD_600_ ~ 0.5, cultures from the test tubes were transferred to petri dishes (the diameter of petri dishes= 100 mm, catalog no. FB0875713, Fisher Scientific). This procedure created a thin film of culture in the dish with a height of about 0.25 mm. Then, these cultures were exposed to a light source emitting UV-B (302 nm) at approximately 2000μW (UVP ChemStudio, catalog no. 849–97-0928–02; Analytik Jena, Jena, Germany) for varying exposure times (0, 2, 4, 8, 16, 24 and 32 min). After UV exposure, cells were transferred back to test tubes and recovered for 24 h. During the recovery period, 10 μl samples were collected at specified time points from each test tube, serially diluted in PBS in round-bottom 96-well plates, and then, plated on LB agar media. The plates were incubated at 37 °C for 16 h to enumerate CFU. We note that new colonies were not formed when incubated beyond 16 h. A similar procedure was followed for the other strains.

### Assessing mutagenesis

To assess the extent of mutant cell formation induced by UV exposure, cells were exposed to UV radiation for the specified durations, followed by a 24-h recovery period. After this recovery period, 500 μL of cells were collected and spread onto agar plates containing 500 μg/mL of RIF. These plates were then incubated at 37 °C for 16 h to enumerate RIF-resistant colonies. To determine clonogenic survival, CFU levels were determined, and mutant levels were normalized by dividing the number of RIF-resistant colonies by the total number of colonies in a 1 mL culture volume. Unless specified otherwise, mutant formation was reported as the count of RIF-resistant colonies per 10^8^ cell population.

### PI Staining

Mid-exponential *E. coli* cells were treated with UV for 16 min and 32 min duration following the protocol mentioned above. At the beginning of the recovery and 1h after the recovery (t=0 h and 1 h), UV-treated cells in LB media were diluted 20-fold in 1.0 ml 0.85% NaCl solution in flow cytometry tubes (5 ml round bottom Falcon tubes, size: 12 × 75 mm) to achieve the desired cell density (~10^6^-10^7^ cells/ml) for flow cytometry analysis. The resulting cell suspensions were treated with 20 μM PI dye. PI produces red fluorescence upon binding DNA; however, it can only penetrate cells with damaged membranes. The samples were incubated in the dark at 37 °C for 15 min before analyzing them with a conventional bench-top flow cytometer (NovoCyte 3000RYB, ACEA Biosciences Inc., San Diego, CA, United States). For flow cytometry analysis, we chose a slow sample flow rate (14 μl/ min) to have a sample stream diameter (i.e., core diameter) of 7.7 μm. The instrument has a constant sheath flow rate of 6.5 ml/min. The flow cytometer utilizes low-power solid-state lasers. Cells were excited at a 561 nm wavelength and red fluorescence was detected with a 615/20-nm bandpass filter. At least 30,000 events were recorded for each sample. NovoExpress software was used to collect the data. PI-stained dead cells, obtained after ethanol (70% v/v) treatment, were used as a positive control. PI-stained live cells were used as a negative control. Forward and side scatter signals of untreated live cells were used to determine the cells on flow cytometry diagrams; the positive and negative controls were used to gate PI positive (+) and PI negative (−) cell populations (Supplementary Fig. S1)

### H_2_O_2_ measurement

The Amplex^™^ Red Hydrogen Peroxide/Peroxidase Assay Kit (Invitrogen, Thermo fischer scientific, Catalog number: A22188) was used to assess the amount of H_2_O_2_ formed in the UV-treated cells. The Amplex^™^ Red Hydrogen Peroxide/Peroxidase Assay Kit contains a sensitive, one-step assay that uses the Amplex^™^ Red reagent (10-acetyl-3,7-dihydroxyphenoxazine) in combination with horseradish peroxidase (HRP) to detect hydrogen peroxide (H_2_O_2_). In the presence of peroxidase, the Amplex^™^ Red reagent reacts with H_2_O_2_ in a 1:1 stoichiometry to produce the red-fluorescent oxidation product, resorufin. Resorufin has excitation and emission maxima of approximately 571 nm and 585 nm. For preparing the stock solutions, 10 mM Amplex^®^ Red reagent was prepared by dissolving the contents of the vial of Amplex^®^ Red reagent in 60 μL of DMSO. Reaction buffer was diluted 5-fold to prepare 1X reaction buffer and 10 U/mL Horseradish Peroxidase (HRP) was prepared by dissolving one vial of HRP in 1.0 mL of 1X Reaction Buffer. The working solution of 100 μM Amplex^®^ Red reagent and 0.2 U/mL HRP was prepared by adding 50 μL of 10 mM Amplex^®^ Red reagent stock solution and 100 μL of 10 U/mL HRP stock solution in 4.85 mL of 1X Reaction Buffer. The assay volume for this experiment was 100 μL. Mid-exponential *E. coli* cells were treated with UV for 16 min and 32 min duration and right after the treatment, 50 μL of sample for each condition was serially diluted in 1X Reaction Buffer to determine the optimal amount of sample for the assay. 50 μL of the Amplex^®^ Red reagent/HRP working solution was added to each microplate well containing the samples. The samples were incubated at room temperature for 30 min, and protected from light. At the end, the fluorescence was measured using a microplate reader equipped for excitation in the range of 530–560 nm and fluorescence emission detection at ~590 nm. For the standard curve, 20 mM Hydrogen Peroxide (H_2_O_2_) working solution was prepared by dissolving 3.0% (0.88 M) H_2_O_2_ in 1X Reaction Buffer and then further diluting 20 mM H_2_O_2_ working solution into 1X Reaction Buffer to produce H_2_O_2_ concentrations of 0 to 10 μM, each in a volume of 50 μL. 50 μL of the Amplex^®^ Red reagent/HRP working solution was added to each microplate well containing the standards followed by 30 min of incubation and then measuring the fluorescence following the same way (Supplementary Fig. S2).

### Fluorescent protein expression assay for reporter genes

Overnight pre-cultures of *E. coli* MG1655 cells with reporter genes fused to the SOS gene promoters (P_*recA*_, P_*uvrA*_, and P_*uvrD*_) were diluted 1:100 in 2 mL of LB media within test tubes and incubated at 37°C with shaking (250 rpm). Upon reaching the mid-exponential phase, the cells were subjected to UV radiation for specified durations (16 min and 32 min). An untreated culture was used as a control. At the beginning of the recovery, 20 μM PI dye was added to the cultures for continuous monitoring of the membrane permeability. At specified time points during the recovery (t=0, 0.25, 0.5, 1, 2, 3 and 4 h), UV-treated cells were diluted 20-fold in 1.0 ml 0.85% NaCl solution in flow cytometry tubes (5 ml round bottom Falcon tubes, size: 12 × 75 mm) to achieve the desired cell density (~10^6^-10^7^ cells/ml). The samples were analyzed using the same flow cytometry method described above (see the “PI staining” section); however, cells were analyzed using two lasers. For measuring the red fluorescence from PI dye, cells were excited at a 561 nm wavelength, and red fluorescence was detected with a 615/20-nm bandpass filter. For measuring the green fluorescence, cells were excited at a 488 nm wavelength, and the green fluorescence was detected with a 530/30 bandpass filter.

### Transcription/translation activities of pUA66-*gfp* plasmid

To assess the effect of the UV treatment on transcription/translation, the amount of GFP produced by *E. coli* strains from the low-copy plasmid was measured. The plasmid, pUA66-*gfp*, has a *gfp* gene under the control of a strong, IPTG-inducible T5 promoter and a strong LacI^q^ repressor. Overnight pre-cultures of *E. coli* MG1655 cells carrying pUA66-*gfp* were diluted 100-fold in 2 mL fresh LB media in test tubes and grown at 37°C with shaking (250 rpm). At the mid-exponential phase (OD_600_~0.5), 0.1mM IPTG was added to the test tubes and then immediately treated with UV for 16 min and 32 min duration. Untreated cultures having IPTG only (without UV exposure) served as controls. At specified time points during the recovery (t=0, 0.25, 0.5, 1, 2, 3, and 4 h), UV-treated cells were diluted 20-fold in 1.0 ml PBS in flow cytometry tubes (5 ml round bottom Falcon tubes, size: 12 × 75 mm) to achieve the desired cell density (~10^6^-10^7^ cells/ml). The samples were analyzed using the same flow cytometry method described above (see the “PI staining” section); however, cells were analyzed with a laser emitting light at 488 nm and the green fluorescence was detected with a 530/30 bandpass filter.

### Promoter library screening

Overnight precultures were prepared by inoculating the strains from the promoter library into the wells of 96-well plates containing 200 μl LB medium and 50 μg/ml kanamycin (for plasmid retention). The plates were sealed with a sterile, oxygen-permeable membrane (Breathe-Easier, Cat# BERM-2000, VWR International) and cultured for 24 h at 37°C with shaking at 250 rpm. Overnight precultures were diluted 1:40 in LB medium with kanamycin in a new 96-well plate, sealed, and incubated at 37°C with shaking at 250 rpm. At the mid-exponential phase (OD_600_ = 0.5), cultures in 96 well plates were exposed to UV light (UVP ChemStudio, catalog no. 849–97-0928–02; Analytik Jena, Jena, Germany) for 16 min, and then, recovered for 24 h at 37°C with shaking at 250 rpm. GFP was measured with a Varioskan LUX Multimode Microplate Reader (Thermo Fisher, Waltham, MA, USA) at the indicated times with untreated cultures as a control. The excitation and emission wavelengths for GFP measurement were 485 nm and 511 nm, respectively. Fold changes for GFP (treated/untreated cultures after 24 h treatment) were used to report the promoter activity. The top 10 promoters showing the highest expression were selected for subsequent experiments.

### Screening *E. coli* BW25113 Keio knockout collection

Overnight cultures of single mutants (Supplementary Table 1) carrying kanamycin resistance genes were diluted 40-fold into flat-bottom 96-well plates, with each well containing 200 μL of cell culture. Kanamycin (50 μg/ml) was added to overnight and treatment cultures to prevent contamination. The plates were securely sealed using a sterile, oxygen-permeable membrane (Breathe-Easier, Cat# BERM-2000, VWR International) and incubated at 37°C with agitation at 250 rpm. When the cultures reached the mid-exponential phase (OD_600_ ~ 0.5), the cells in the 96-well plates were exposed to UV light (UVP ChemStudio, catalog no. 849–97-0928–02; Analytik Jena, Jena, Germany) for 16 min. Subsequently, they were allowed to recover for 24 h at 37°C with shaking at 250 rpm. To quantify UV-induced mutant cells, 10 μL samples were collected from each well and spotted onto LB agar plates, containing 500 μg/mL RIF, at the end of the 24-h recovery period. The plates were then incubated at 37°C for 16 h to enumerate RIF-resistant colonies. The mutant formation was reported as the count of RIF-resistant colonies per 10 μL of cell culture.

### Statistical analysis and reproducibility

For all pairwise comparisons, one-way ANOVA with Dunnett’s post-test was utilized. A minimum of four independent biological replicates (unless otherwise specified) were conducted for experiments involving UV exposure. In all figures, data corresponding to each time point represents the mean value ± standard deviation. Regarding statistical significance analysis, the threshold values were set as follows: *P < 0.05, **P < 0.01, ***P < 0.001, and ****P < 0.0001. All figures were created using GraphPad Prism 10.0.2, and the statistical analyses were carried out using GraphPad Prism 10.0.2 statistical functions. FlowJo V 10.7.1 was used to analyze the data obtained from flow cytometry.

## Supplementary Material

1

## Data Availability

All data in this manuscript are available in either the main text or supplementary files. All raw data have been deposited in the Figshare repository (doi.org/10.6084/m9.figshare.27701493.v1).

## References

[1] Hershberg R. Mutation—The Engine of Evolution: Studying Mutation and Its Role in the Evolution of Bacteria. Cold Spring Harb Perspect Biol 7, (2015). 10.1101/cshperspect.a018077 PMC4563715 26330518

[2] MacLean R. C., Torres-Barceló C. & Moxon R. Evaluating evolutionary models of stress-induced mutagenesis in bacteria. Nature Reviews Genetics 2013 14:3 14, 221–227 (2013). 10.1038/nrg3415 23400102

[3] Zimmermann A., Prieto-Vivas J. E., Voordeckers K., Bi C. & Verstrepen K. J. Mutagenesis techniques for evolutionary engineering of microbes – exploiting CRISPR-Cas, oligonucleotides, recombinases, and polymerases. Trends Microbiol (2024) doi:10.1016/J.TIM.2024.02.006. 38493013

[4] Sung W., Ackerman M. S., Miller S. F., Doak T. G. & Lynch M. Drift-barrier hypothesis and mutation-rate evolution. Proc Natl Acad Sci U S A 109, 18488–18492 (2012). 23077252 10.1073/pnas.1216223109 PMC3494944

[5] Wielgoss S. Mutation rate dynamics in a bacterial population reflect tension between adaptation and genetic load. Proc Natl Acad Sci U S A 110, 222–227 (2013). 23248287 10.1073/pnas.1219574110 PMC3538217

[6] Fitzgerald D. M., Hastings P. J. & Rosenberg S. M. Stress-Induced Mutagenesis: Implications in Cancer and Drug Resistance. Annu Rev Cancer Biol 1, 119 (2017). 29399660 10.1146/annurev-cancerbio-050216-121919 PMC5794033

[7] Morreall J. F., Petrova L. & Doetsch P. W. Transcriptional mutagenesis and its potential roles in the etiology of cancer and bacterial antibiotic resistance. J Cell Physiol 228, 2257 (2013). 23696333 10.1002/jcp.24400 PMC3963475

[8] Russo M., Sogari A. & Bardelli A. Adaptive evolution: How bacteria and cancer cells survive stressful conditions and drug treatment. Cancer Discov 11, 1886–1895 (2021). 33952585 10.1158/2159-8290.CD-20-1588

[9] Lambert G. An analogy between the evolution of drug resistance in bacterial communities and malignant tissues. Nat Rev Cancer 11, 375 (2011). 21508974 10.1038/nrc3039 PMC3488437

[10] Rothschild L. J. The Influence of UV Radiation on Protistan Evolution. Journal of Eukaryotic Microbiology 46, 548–555 (1999). 11536924 10.1111/j.1550-7408.1999.tb06074.x

[11] Rozema J. The role of UV-B radiation in aquatic and terrestrial ecosystems—an experimental and functional analysis of the evolution of UV-absorbing compounds. J Photochem Photobiol B 66, 2–12 (2002). 11849977 10.1016/s1011-1344(01)00269-x

[12] Rastogi R. P., Richa, Kumar A., Tyagi M. B. & Sinha R. P. Molecular Mechanisms of Ultraviolet Radiation-Induced DNA Damage and Repair. J Nucleic Acids 2010, 32 (2010). 10.4061/2010/592980 PMC3010660 21209706

[13] Sinha R. P. & Häder D. P. UV-induced DNA damage and repair: a review. Photochemical & Photobiological Sciences 2002 1:4 1, 225–236 (2002). 10.1039/b201230h 12661961

[14] Krishna S., Maslov S. & Sneppen K. UV-Induced Mutagenesis in Escherichia coli SOS Response: A Quantitative Model. PLoS Comput Biol 3, e41 (2007). 17367202 10.1371/journal.pcbi.0030041 PMC1828700

[15] Kciuk M., Marciniak B., Mojzych M. & Kontek R. Focus on UV-Induced DNA Damage and Repair—Disease Relevance and Protective Strategies. Int J Mol Sci 21, 1–33 (2020). 10.3390/ijms21197264 PMC7582305 33019598

[16] Gray N. F. Ultraviolet Disinfection. Microbiology of Waterborne Diseases: Microbiological Aspects and Risks: Second Edition 617–630 (2014) doi:10.1016/B978-0-12-415846-7.00034-2.

[17] Hessling M., Haag R., Sieber N. & Vatter P. The impact of far-UVC radiation (200–230 nm) on pathogens, cells, skin, and eyes - a collection and analysis of a hundred years of data. GMS Hyg Infect Control 16, Doc07 (2021). 33643774 10.3205/dgkh000378 PMC7894148

[18] Black H. S. Photocarcinogenesis: an overview. J Photochem Photobiol B 40, 29–47 (1997). 9301042 10.1016/s1011-1344(97)00021-3

[19] Douki T., Court M., Sauvaigo S., Odin F. & Cadet J. Formation of the main UV-induced thymine dimeric lesions within isolated and cellular DNA as measured by high performance liquid chromatography-tandem mass spectrometry. J Biol Chem 275, 11678–11685 (2000). 10766787 10.1074/jbc.275.16.11678

[20] Rupert C. S. Enzymatic Photoreactivation: Overview. Basic Life Sci 5 A, 73–87 (1975). 10.1007/978-1-4684-2895-7_11 1103856

[21] Seawell P. C. den V gene of bacteriophage T4 determines a DNA glycosylase specific for pyrimidine dimers in DNA. J Virol 35, 790–796 (1980). 6252338 10.1128/jvi.35.3.790-796.1980 PMC288873

[22] Van Houten B. Nucleotide excision repair in Escherichia coli. Microbiol Rev 54, 18–51 (1990). 2181258 10.1128/mr.54.1.18-51.1990 PMC372757

[23] Rupp W. D. & Howard-flanders P. Discontinuities in the DNA synthesized in an Excision-defective strain of Escherichia coli following ultraviolet irradiation. J Mol Biol 31, 291–304 (1968). 4865486 10.1016/0022-2836(68)90445-2

[24] Wendel B. M., Hollingsworth S., Courcelle C. T. & Courcelle J. UV-induced DNA damage disrupts the coordination between replication initiation, elongation and completion. Genes to Cells 26, 94–108 (2021). 33382157 10.1111/gtc.12826

[25] Majchrzak M., Bowater R. P., Staczek P. & Parniewski P. SOS Repair and DNA Supercoiling Influence the Genetic Stability of DNA Triplet Repeats in Escherichia coli. J Mol Biol 364, 612–624 (2006). 17028021 10.1016/j.jmb.2006.08.093

[26] Simmons L. A., Foti J. J., Cohen S. E. & Walker G. C. The SOS Regulatory Network. EcoSal Plus 3, (2008). 10.1128/ecosalplus.5.4.3 26443738

[27] Little J. W. & Mount D. W. The SOS regulatory system of Escherichia coli. Cell 29, 11–22 (1982). 7049397 10.1016/0092-8674(82)90085-x

[28] Maslowska K. H., Makiela-Dzbenska K. & Fijalkowska I. J. The SOS system: A complex and tightly regulated response to DNA damage. Environ Mol Mutagen 60, 368–384 (2019). 30447030 10.1002/em.22267 PMC6590174

[29] Walker G. C. Mutagenesis and inducible responses to deoxyribonucleic acid damage in Escherichia coli. Microbiol Rev 48, 60–93 (1984). 6371470 10.1128/mr.48.1.60-93.1984 PMC373003

[30] Montelone B. A. DNA Repair and Mutagenesis. Second Edition. By Friedberg Errol C, Walker Graham C, Siede Wolfram, Wood Richard D, Schultz Roger A, and, Ellenberger Tom. Washington (DC): ASM Press. $179.95. xxix + 1118 p + 11 pl; ill.; index. ISBN: 1–55581-319–4. 2006.. 10.1086/509407 81, 273–273 (2015).

[31] Martins-Pinheiro M., Marques R. C. P. & Menck C. F. M. Genome analysis of DNA repair genes in the alpha proteobacterium Caulobacter crescentus. BMC Microbiol 7, 17 (2007). 17352799 10.1186/1471-2180-7-17 PMC1839093

[32] Kuzminov A. Recombinational Repair of DNA Damage in Escherichia coli and Bacteriophage λ. Microbiology and Molecular Biology Reviews 63, 751–813 (1999). 10585965 10.1128/mmbr.63.4.751-813.1999 PMC98976

[33] Burckhardt S. E., Woodgate R., Scheuermann R. H. & Echols H. UmuD mutagenesis protein of Escherichia coli: overproduction, purification, and cleavage by RecA. Proceedings of the National Academy of Sciences 85, 1811–1815 (1988). 10.1073/pnas.85.6.1811 PMC279869 3279417

[34] Woodgate R., Rajagopalan M., Lu C. & Echols H. UmuC mutagenesis protein of Escherichia coli: purification and interaction with UmuD and UmuD’. Proceedings of the National Academy of Sciences 86, 7301–7305 (1989). 10.1073/pnas.86.19.7301 PMC298049 2552436

[35] Kato T. & Shinoura Y. Isolation and characterization of mutants of Escherichia coli deficient in induction of mutations by ultraviolet light. MGG Molecular & General Genetics 156, 121–131 (1977). 340898 10.1007/BF00283484

[36] Wang Z. Translesion synthesis by the UmuC family of DNA polymerases. Mutation Research/DNA Repair 486, 59–70 (2001). 11425512 10.1016/s0921-8777(01)00089-1

[37] Liu J. MutL sliding clamps coordinate exonuclease-independent Escherichia coli mismatch repair. Nat Commun 10, (2019). 10.1038/s41467-019-13191-5 PMC6876574 31757945

[38] Lin T. Contribution of the SOS response and the DNA repair systems to norfloxacin induced mutations in E. coli. Mar Life Sci Technol 5, 538–550 (2023). 38045542 10.1007/s42995-023-00185-y PMC10689325

[39] Shibai A. Mutation accumulation under UV radiation in Escherichia coli. Scientific Reports 2017 7:1 7, 1–12 (2017). 10.1038/s41598-017-15008-1 PMC5674018 29109412

[40] Badran A. H. & Liu D. R. Development of potent in vivo mutagenesis plasmids with broad mutational spectra. Nature Communications 2015 6:1 6, 1–10 (2015). 10.1038/ncomms9425 PMC4633624 26443021

[41] Tillich U. M., Lehmann S., Schulze K., Dühring U. & Frohme M. The Optimal Mutagen Dosage to Induce Point-Mutations in Synechocystis sp. PCC6803 and Its Application to Promote Temperature Tolerance. PLoS One 7, e49467 (2012). 23185339 10.1371/journal.pone.0049467 PMC3504032

[42] Hashimoto S. Isolation of auxotrophic mutants of diploid industrial yeast strains after UV mutagenesis. Appl Environ Microbiol 71, 312–319 (2005). 15640203 10.1128/AEM.71.1.312-319.2005 PMC544216

[43] Hijnen W. A. M., Beerendonk E. F. & Medema G. J. Inactivation credit of UV radiation for viruses, bacteria and protozoan (oo)cysts in water: A review. Water Res 40, 3–22 (2006). 16386286 10.1016/j.watres.2005.10.030

[44] Reed N. G. The History of Ultraviolet Germicidal Irradiation for Air Disinfection. Public Health Reports 125, 15 (2010). 10.1177/003335491012500105 PMC2789813 20402193

[45] Bergkessel M., Basta D. W. & Newman D. K. The physiology of growth arrest: uniting molecular and environmental microbiology. Nat Rev Microbiol 14, 549 (2016). 27510862 10.1038/nrmicro.2016.107 PMC10069271

[46] Dörr T., Vulić M. & Lewis K. Ciprofloxacin causes persister formation by inducing the TisB toxin in Escherichia coli. PLoS Biol 8, 29–35 (2010). 10.1371/journal.pbio.1000317 PMC2826370 20186264

[47] Völzing K. G. & Brynildsen M. P. Stationary-phase persisters to ofloxacin sustain DNA damage and require repair systems only during recovery. mBio 6, (2015). 10.1128/mBio.00731-15 PMC4556807 26330511

[48] Santos A. L. Effects of UV radiation on the lipids and proteins of bacteria studied by mid-infrared spectroscopy. Environ Sci Technol 47, 6306–6315 (2013). 23692317 10.1021/es400660g

[49] Wurtmann E. J. & Wolin S. L. RNA under attack: Cellular handling of RNA damage. Crit Rev Biochem Mol Biol 44, 34 (2009). 19089684 10.1080/10409230802594043 PMC2656420

[50] Ghosh S. & Orman M. A. Exploring the links between SOS response, mutagenesis, and resistance during the recovery period. Antimicrob Agents Chemother 68, (2024). 10.1128/aac.01462-23 PMC11064565 38534113

[51] Barrett T. C., Mok W. W. K., Murawski A. M. & Brynildsen M. P. Enhanced antibiotic resistance development from fluoroquinolone persisters after a single exposure to antibiotic. Nature Communications 2019 10:1 10, 1–11 (2019). 10.1038/s41467-019-09058-4 PMC6414640 30862812

[52] Krašovec R. Mutation rate plasticity in rifampicin resistance depends on Escherichia coli cell-cell interactions. Nat Commun 5, (2014). 10.1038/ncomms4742 PMC4007418 24776982

[53] Reynolds M. G. Compensatory evolution in rifampin-resistant Escherichia coli. Genetics 156, 1471 (2000). 11102350 10.1093/genetics/156.4.1471 PMC1461348

[54] Pope C. F., O’Sullivan D. M., McHugh T. D. & Gillespie S. H. A Practical Guide to Measuring Mutation Rates in Antibiotic Resistance. Antimicrob Agents Chemother 52, 1209 (2008). 18250188 10.1128/AAC.01152-07 PMC2292516

[55] Rosche W. A. & Foster P. L. Determining Mutation Rates in Bacterial Populations. Methods 20, 4 (2000). 10610800 10.1006/meth.1999.0901 PMC2932672

[56] Lagage V., Chen V. & Uphoff S. Adaptation delay causes a burst of mutations in bacteria responding to oxidative stress. EMBO Rep 24, (2023). 10.15252/embr.202255640 PMC9827559 36397732

[57] Rodríguez-Rojas A. Non-lethal exposure to H2O2 boosts bacterial survival and evolvability against oxidative stress. PLoS Genet 16, e1008649 (2020). 32163413 10.1371/journal.pgen.1008649 PMC7093028

[58] Zaslaver A. A comprehensive library of fluorescent transcriptional reporters for Escherichia coli. Nat Methods 3, 623–628 (2006). 16862137 10.1038/nmeth895

[59] Keseler I. M. The EcoCyc database: reflecting new knowledge about Escherichia coli K-12. Nucleic Acids Res 45, D543–D550 (2017). 27899573 10.1093/nar/gkw1003 PMC5210515

[60] Fernández De Henestrosa A. R. Identification of additional genes belonging to the LexA regulon in Escherichia coli. Mol Microbiol 35, 1560–1572 (2000). 10760155 10.1046/j.1365-2958.2000.01826.x

[61] Goodall E. C. A. The Essential Genome of Escherichia coli K-12. mBio 9, (2018). 10.1128/mBio.02096-17 PMC5821084 29463657

[62] Yu X.-C., Weihe E. K. & Margolin W. Role of the C Terminus of FtsK in Escherichia coli Chromosome Segregation. J Bacteriol 180, 6424 (1998). 9829960 10.1128/jb.180.23.6424-6428.1998 PMC107737

[63] Wang L. & Lutkenhaus J. FtsK is an essential cell division protein that is localized to the septum and induced as part of the SOS response. Mol Microbiol 29, 731–740 (1998). 9723913 10.1046/j.1365-2958.1998.00958.x

[64] Meddows T. R., Savory A. P., Grove J. I., Moore T. & Lloyd R. G. RecN protein and transcription factor DksA combine to promote faithful recombinational repair of DNA double-strand breaks. Mol Microbiol 57, 97–110 (2005). 15948952 10.1111/j.1365-2958.2005.04677.x

[65] Picksley S. M., Attfield P. V. & Lloyd R. G. Repair of DNA double-strand breaks in Escherichia coli K12 requires a functional recN product. Mol Gen Genet 195, 267–274 (1984). 6092851 10.1007/BF00332758

[66] Wang T. C. & Smith K. C. Different effects of recJ and recN mutations on the postreplication repair of UV-damaged DNA in Escherichia coli K-12. J Bacteriol 170, 2555–2559 (1988). 3286613 10.1128/jb.170.6.2555-2559.1988 PMC211171

[67] Kosinski J., Feder M. & Bujnicki J. M. The PD-(D/E)XK superfamily revisited: identification of new members among proteins involved in DNA metabolism and functional predictions for domains of (hitherto) unknown function. BMC Bioinformatics 6, (2005). 10.1186/1471-2105-6-172 PMC1189080 16011798

[68] Lomize A. L., Lomize M. A., Krolicki S. R. & Pogozheva I. D. Membranome: a database for proteome-wide analysis of single-pass membrane proteins. Nucleic Acids Res 45, D250–D255 (2017). 27510400 10.1093/nar/gkw712 PMC5210604

[69] Napolitano R., Janel-Bintz R., Wagner J. & Fuchs R. P. P. All three SOS-inducible DNA polymerases (Pol II, Pol IV and Pol V) are involved in induced mutagenesis. EMBO J 19, 6259 (2000). 11080171 10.1093/emboj/19.22.6259 PMC305832

[70] Hickson I. D., Robson C. N., Atkinson K. E., Hutton L. & Emmerson P. T. Reconstitution of RecBC DNase activity from purified Escherichia coli RecB and RecC proteins. J Biol Chem 260, 1224–9 (1985). 3155726

[71] Payne-Dwyer A. L., Syeda A. H., Shepherd J. W., Frame L. & Leake M. C. RecA and RecB: probing complexes of DNA repair proteins with mitomycin C in live Escherichia coli with single-molecule sensitivity. J R Soc Interface 19, (2022). 10.1098/rsif.2022.0437 PMC9363994 35946163

[72] Reuven N. B., Arad G., Maor-Shoshani A. & Livneh Z. The mutagenesis protein UmuC is a DNA polymerase activated by UmuD’, RecA, and SSB and is specialized for translesion replication. J Biol Chem 274, 31763–31766 (1999). 10542196 10.1074/jbc.274.45.31763

[73] Takahagi M., Iwasaki H., Nakata A. & Shinagawa H. Molecular analysis of the Escherichia coli ruvC gene, which encodes a Holliday junction-specific endonuclease. J Bacteriol 173, 5747–5753 (1991). 1885548 10.1128/jb.173.18.5747-5753.1991 PMC208306

[74] Loewen P. C. Isolation of catalase-deficient Escherichia coli mutants and genetic mapping of katE, a locus that affects catalase activity. J Bacteriol 157, 622–626 (1984). 6319370 10.1128/jb.157.2.622-626.1984 PMC215291

[75] Epshtein V. UvrD facilitates DNA repair by pulling RNA polymerase backwards. Nature 505, 372–377 (2014). 24402227 10.1038/nature12928 PMC4471481

[76] Orren D. K. & Sancar A. The (A)BC excinuclease of Escherichia coli has only the UvrB and UvrC subunits in the incision complex. Proc Natl Acad Sci U S A 86, 5237–5241 (1989). 2546148 10.1073/pnas.86.14.5237 PMC297596

[77] de Oliveira A. H. S., da Silva A. E., de Oliveira I. M., Henriques J. A. P. & Agnez-Lima L. F. MutY-glycosylase: An overview on mutagenesis and activities beyond the GO system. Mutation Research/Fundamental and Molecular Mechanisms of Mutagenesis 769, 119–131 (2014). 25771731 10.1016/j.mrfmmm.2014.08.002

[78] Shurtleff B. W., Ollivierre J. N., Tehrani M., Walker G. C. & Beuning P. J. Steric Gate Variants of UmuC Confer UV Hypersensitivity on Escherichia coli. J Bacteriol 191, 4815 (2009). 19482923 10.1128/JB.01742-08 PMC2715726

[79] Wang T. chien V. & Smith, K. C. Role of the umuC gene in postreplication repair in UV-irradiated Escherichia coli K-12 uvrB. Mutation Research/DNA Repair Reports 145, 107–112 (1985). 10.1016/0167-8817(85)90016-1 2984560

[80] Moolenaar G. F., Moorman C. & Goosen N. Role of the Escherichia coli Nucleotide Excision Repair Proteins in DNA Replication. J Bacteriol 182, 5706 (2000). 11004168 10.1128/jb.182.20.5706-5714.2000 PMC94691

[81] Orren D., Selby C., Hearst J. & Sancar A. Post-incision steps of nucleotide excision repair in Escherichia coli. Disassembly of the UvrBC-DNA complex by helicase II and DNA polymerase I. J Biol Chem 267, 780–788 (1992). 1530937

[82] Bertrand-Burggraf E., Selby C. P., Hearst J. E. & Sancar A. Identification of the different intermediates in the interaction of (A)BC excinuclease with its substrates by DNase I footprinting on two uniquely modified oligonucleotides. J Mol Biol 219, 27–36 (1991). 2023258 10.1016/0022-2836(91)90854-y

[83] Higashitani A., Ishii Y., Kato Y. & Horiuchi K. Functional dissection of a cell-division inhibitor, SulA, of Escherichia coli and its negative regulation by Lon. Molecular and General Genetics 254, 351–357 (1997). 9180687 10.1007/s004380050426

[84] Unoson C. & Wagner E. G. H. A small SOS-induced toxin is targeted against the inner membrane in Escherichia coli. Mol Microbiol 70, 258–270 (2008). 18761622 10.1111/j.1365-2958.2008.06416.x

[85] McKenzie K. The effects of 405 nm light on bacterial membrane integrity determined by salt and bile tolerance assays, leakage of UV-absorbing material and SYTOX green labelling. Microbiology (N Y) 162, 1680 (2016). 10.1099/mic.0.000350 PMC5068139 27499074

[86] Yeo S. K. & Liong M. T. Effects and applications of sub-lethal ultrasound, electroporation and UV radiations in bioprocessing. Ann Microbiol 63, 813–824 (2013).

[87] Cadet J., Douki T. & Ravanat J. L. Oxidatively generated base damage to cellular DNA. Free Radic Biol Med 49, 9–21 (2010). 20363317 10.1016/j.freeradbiomed.2010.03.025

[88] Winterbourn C. C. Reconciling the chemistry and biology of reactive oxygen species. Nature Chemical Biology 2008 4:5 4, 278–286 (2008). 10.1038/nchembio.85 18421291

[89] Schieber M. & Chandel N. S. ROS Function in Redox Signaling and Oxidative Stress. Curr Biol 24, R453 (2014). 24845678 10.1016/j.cub.2014.03.034 PMC4055301

[90] Zwieb C., Ross A., Rinke J., Meinke M. & Brimacombe R. Evidence for RNA-RNA cross-link formation in Escherichia coli ribosomes. Nucleic Acids Res 5, 2705 (1978). 358146 10.1093/nar/5.8.2705 PMC342201

[91] Orman M. A. & Brynildsen M. P. Inhibition of stationary phase respiration impairs persister formation in E. coli. Nature Communications 2015 6:1 6, 1–13 (2015). 10.1038/ncomms8983 PMC4530465 26246187

[92] Mohiuddin S. G., Nguyen T. V. & Orman M. A. Pleiotropic actions of phenothiazine drugs are detrimental to Gram-negative bacterial persister cells. Communications Biology 2022 5:1 5, 1–15 (2022). 10.1038/s42003-022-03172-8 PMC8907348 35264714

[93] Mohiuddin S. G., Massahi A. & Orman M. A. lon Deletion Impairs Persister Cell Resuscitation in Escherichia coli. mBio 13, (2022). 10.1128/mbio.02187-21 PMC8764555 35038905

[94] Datsenko K. A. & Wanner B. L. One-step inactivation of chromosomal genes in Escherichia coli K-12 using PCR products. Proc Natl Acad Sci U S A 97, 6640 (2000). 10829079 10.1073/pnas.120163297 PMC18686

