# Supplementary material for: UV-B-Induced DNA Repair Mechanisms and Their Effects on Mutagenesis and Culturability in Escherichia coli
Source: bioRxiv. 2025 Jun 9:2024.11.14.623584. Originally published 2024 Nov 14. Preprint. [Version 2] doi: 10.1101/2024.11.14.623584 (PMC11601333; doi:10.1101/2024.11.14.623584)
Supplement: Supplement 1 [file media-1.pdf]

Supplementary Information

UV-Induced DNA Repair Mechanisms and Their Effects on Mutagenesis and Culturability in *Escherichia coli*

Sreyashi Ghosh[1], Jenet Narzary, and Mehmet A. Orman[1]*
[1]William A. Brookshire Department of Chemical and Biomolecular Engineering, University of Houston, Houston, TX, USA

*Correspondence to: S222 Engineering Bldg 1, 4226 Martin Luther King Boulevard, Houston, TX 77204-4004, Phone: 713-743-6785, Email: morman@central.uh.edu

Supplementary figures

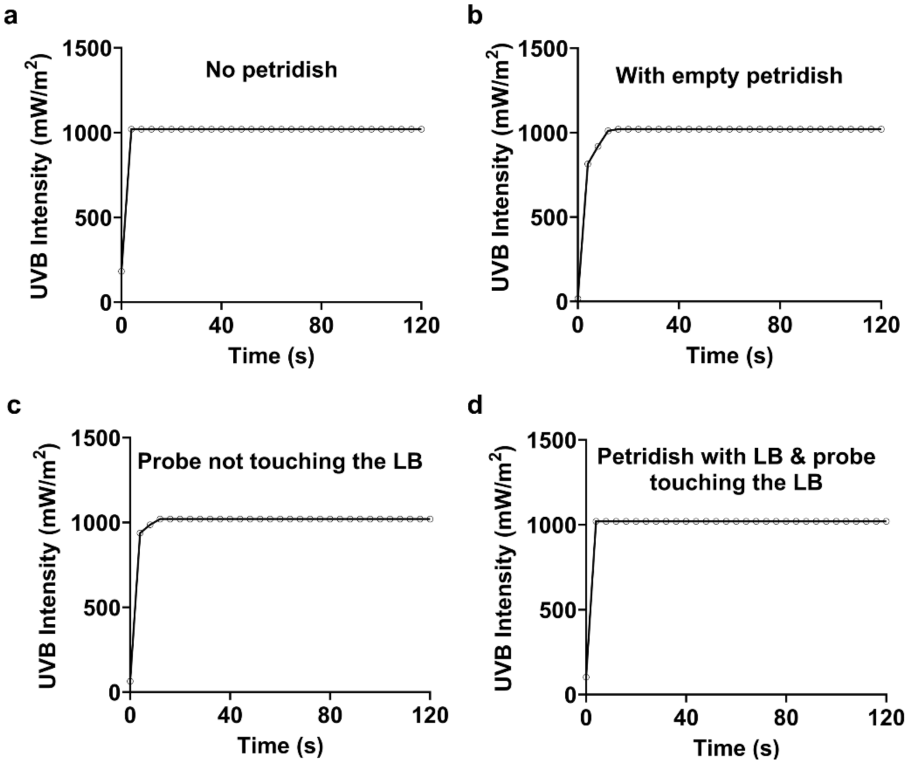

Fig. S1. UV lamp intensity measurement at 302 nm using a UV-B dosimeter. UV-B intensity was measured using a dosimeter (see Materials and Methods for specifications) under four different conditions to ensure accurate assessment of the irradiance reaching bacterial cells. (a) Probe placed directly on the surface of the UV transilluminator block with no Petri dish. (b) Probe placed on an empty Petri dish positioned on the UV transilluminator. (c) Probe positioned inside a Petri dish containing LB medium, without contacting the liquid surface. (d) Probe in direct contact with the LB medium inside the Petri dish on the UV transilluminator block.

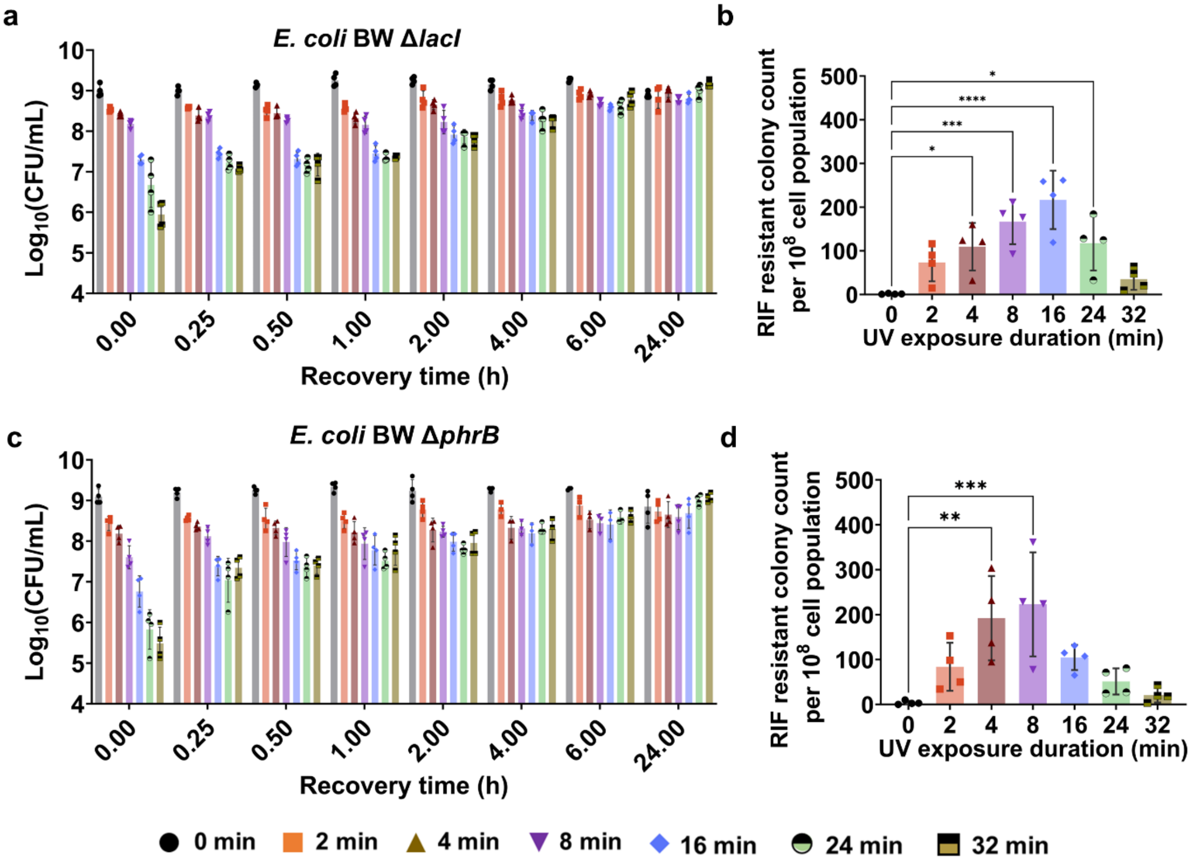

Fig. S2. Photoreactivation-deficient strain *E. coli* K-12 BW25113 Δ*phrB* exhibited similar trends in survival and mutagenesis compared to the Δ*lacI* control strain. (a, c) Exponential-phase *E. coli* BW25113 Δ*lacI* and Δ*phrB* cells were exposed to UV-B radiation for 0, 2, 4, 8, 16, 24, and 32 minutes, followed by a 24-hour recovery period. At designated time points, cells were collected and plated to determine CFU levels. (b, d) UV-induced mutagenesis was assessed by quantifying RIF-resistant colonies (per 10^8 cells) in each knockout strain after recovery at the indicated UV exposure times. Δ*lacI* was used as a reference control. n=4. Statistical analysis was performed using one-way ANOVA with Dunnett's post-test, where $*P < 0.05$, $**P < 0.001$, $***P < 0.01$, $****P < 0.0001$. Data corresponding to each time point represent mean value ± standard deviation.

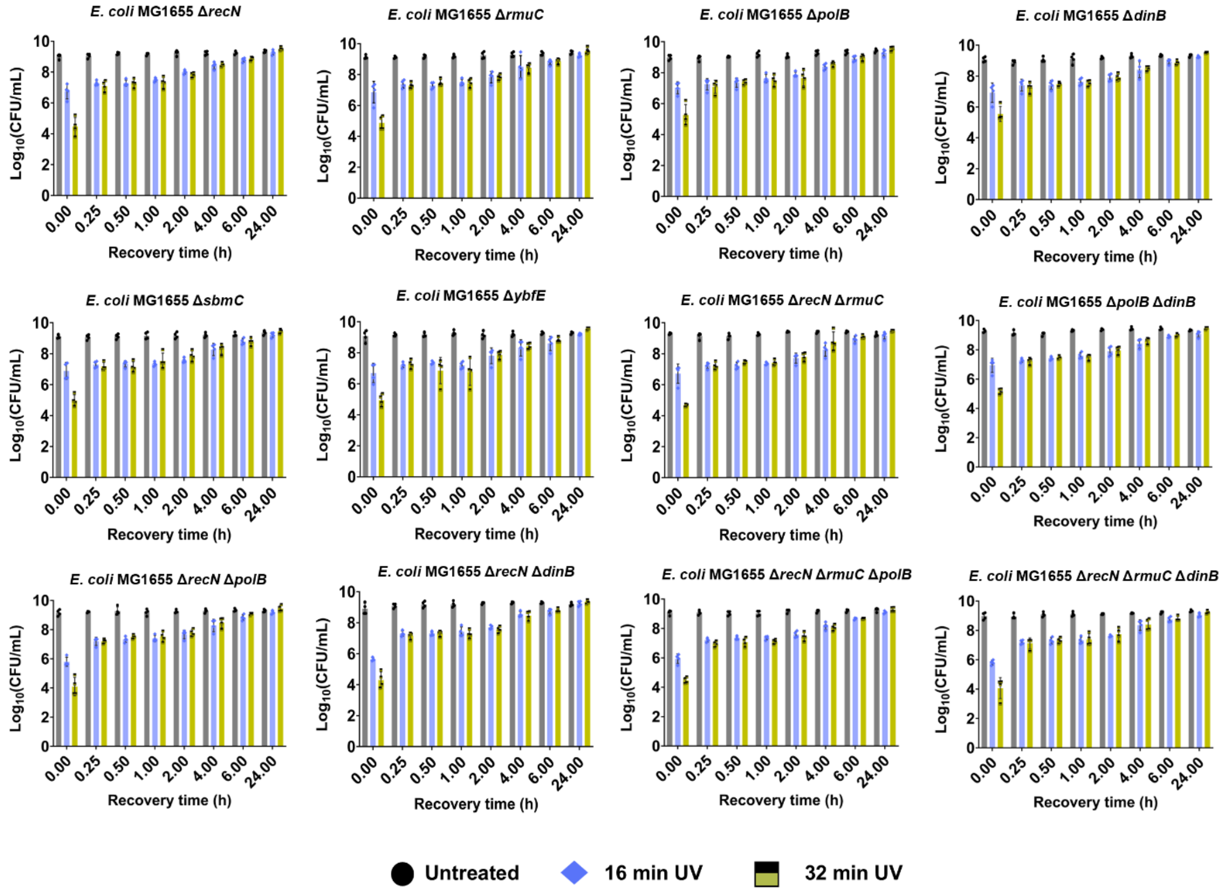

Fig. S3. Assessment of cell culturability over 24 hours in *E. coli* MG1655 strains lacking UV-upregulated genes identified in the promoter screening (Fig. 2a). Genes that were upregulated following UV exposure in the MG1655 promoter-reporter library were individually or combinatorially deleted, and UV-induced mutagenesis assays were performed in the resulting knockout strains. Mid-exponential phase cells were exposed to UV-B for 0, 16, or 32 minutes and allowed to recover for 24 hours. At specified recovery time points (0, 0.25, 0.5, 1, 2, 4, 6, and 24 h), samples were collected and plated to assess CFU. n = 4. Data represent mean ± standard deviation for each time point.

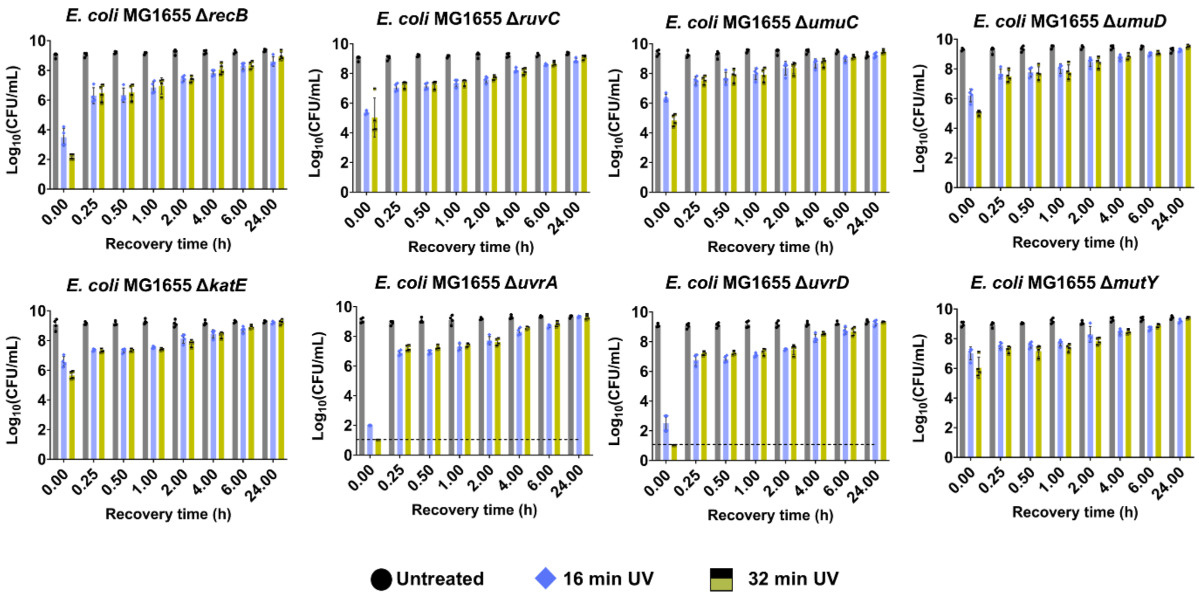

Fig. S4. Assessment of cell culturability over 24 hours in *E. coli* MG1655 strains lacking selected genes from knockout library screening (Fig. 3a). Selected genes identified in the *E. coli* BW25113 single-gene deletion screen were individually deleted in the MG1655 background, and UV-induced mutagenesis assays were performed. Mid-exponential phase cells were exposed to UV-B for 0, 16, or 32 minutes, followed by recovery for 24 hours. At designated time points (0, 0.25, 0.5, 1, 2, 4, 6, and 24 h), samples were collected and plated to assess CFU. n = 4. Data represent the mean ± standard deviation at each time point.

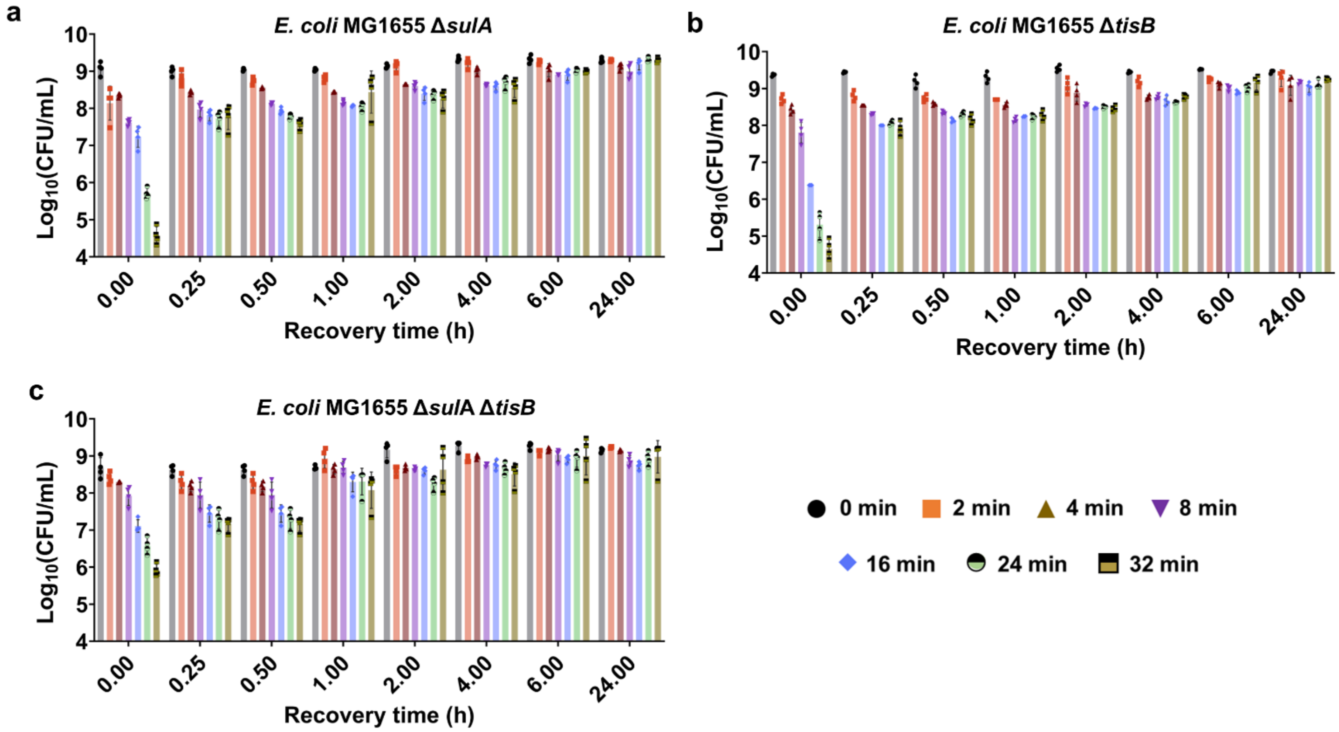

Fig. S5. Assessment of cell culturability over 24 hours in *E. coli* MG1655 strains lacking SulA and TisB (Fig. 4a-c). Mid-exponential-phase *E. coli* MG1655 Δ*sulA*, Δ*tisB*, and Δ*sulA*Δ*tisB* cells were exposed to UV-B light for 0, 2, 4, 8, 16, 24, and 32 minutes, followed by a 24-h recovery period. At designated time points (0, 0.25, 0.5, 1, 2, 4, 6, and 24 h), samples were collected and plated to assess CFU. n = 4. Data represents the mean ± standard deviation at each time point.

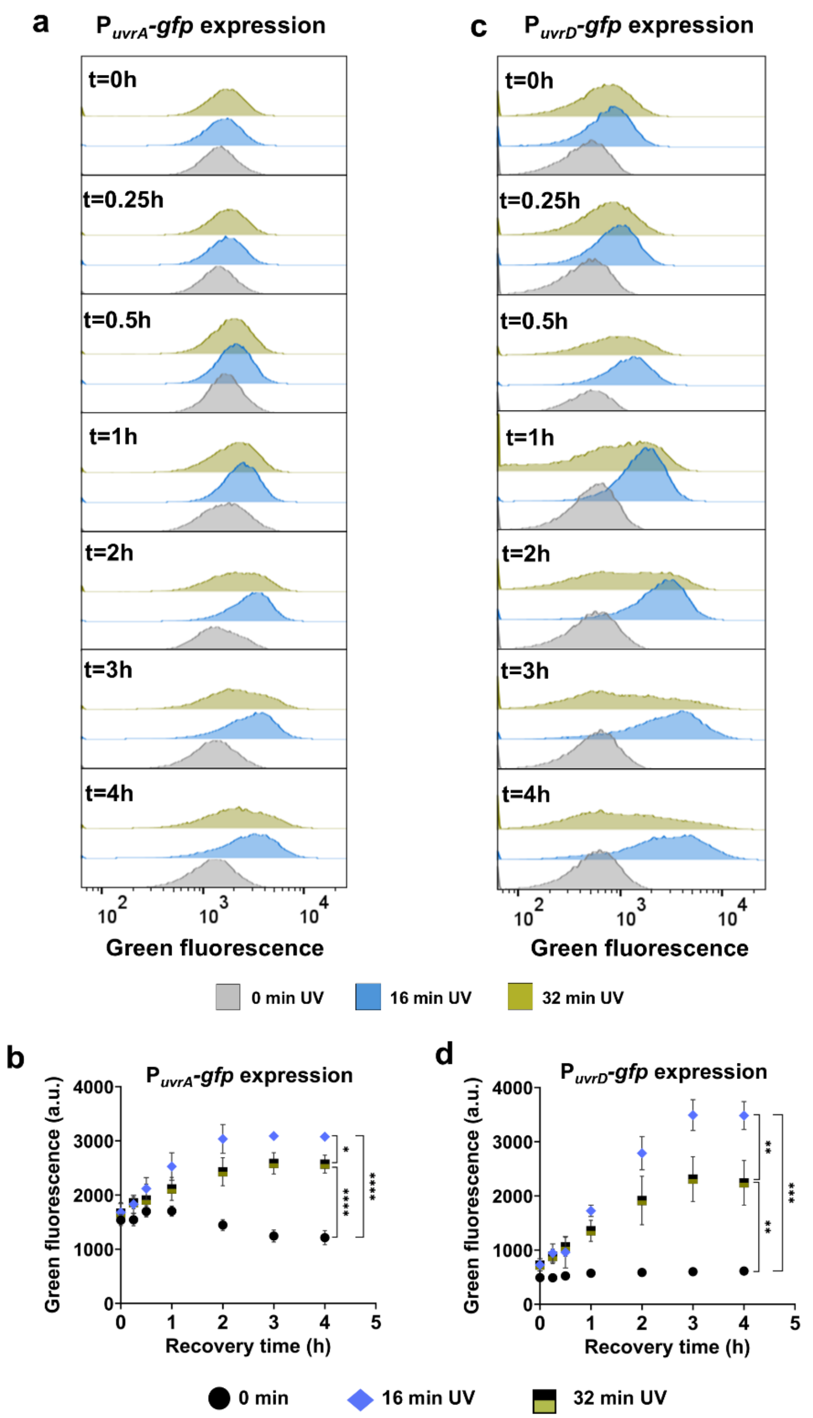

Fig. S6: Flow cytometry analysis of nucleotide excision repair genes followed by UV-treatment (a, b) The GFP profiles of UV-treated *E. coli* MG1655 cells harboring *pMSs201* P_{uvrA}-*gfp*, and (c, d) the GFP profiles of UV-treated *E. coli* MG1655 cells harboring *pMSs201* P_{uvrD}-*gfp* were assessed using flow cytometry after UV exposure (for the indicated time points during recovery). n=4. Statistical analysis was performed using two-way ANOVA, where $*P < 0.05$, $**P < 0.001$, $***P < 0.01$, $****P < 0.0001$. Data corresponding to each time point represent mean value ± standard deviation.

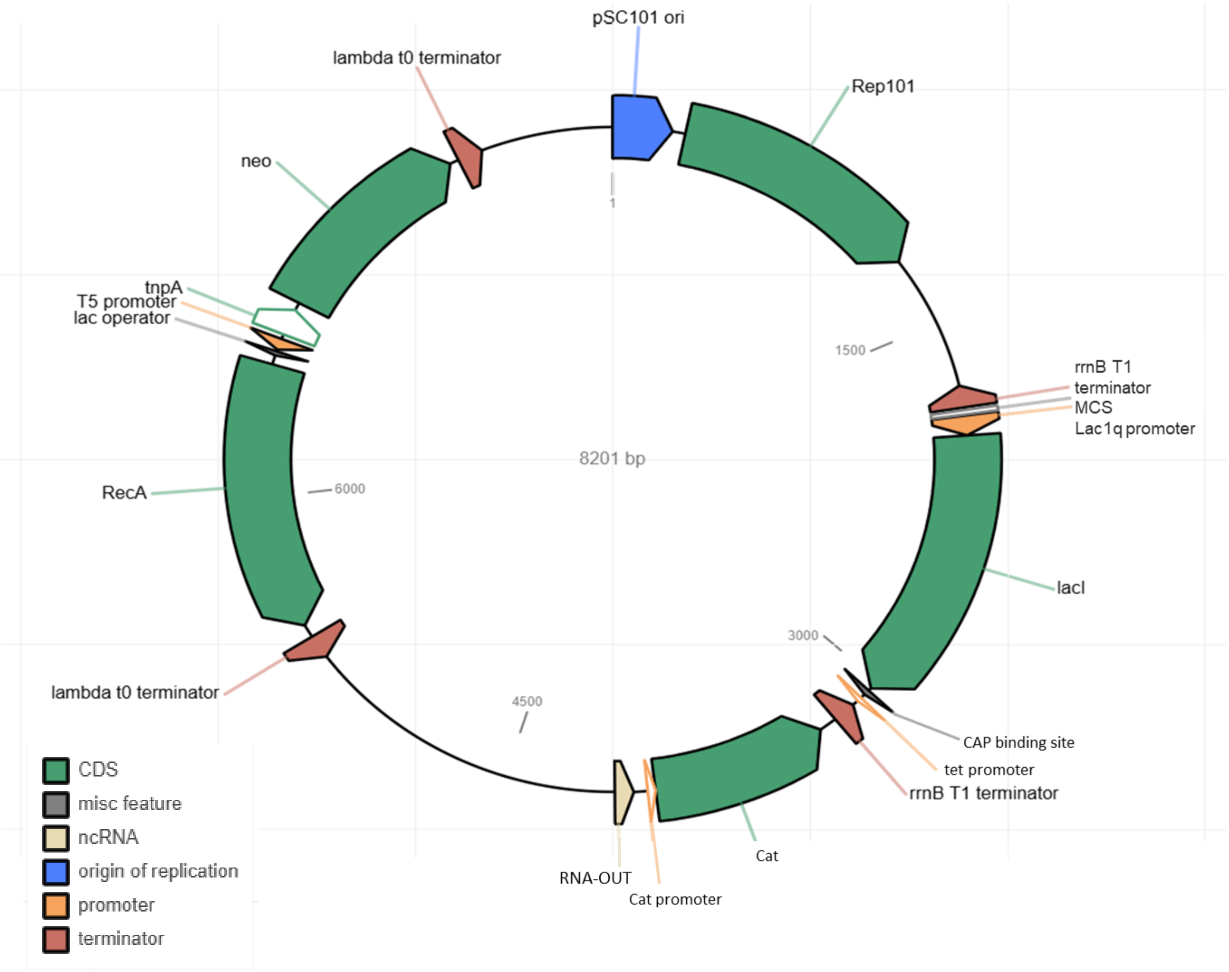

Fig. S7. Annotated plasmid map of the overexpression plasmid pUA66-*recA*. An IPTG-inducible *recA* overexpression plasmid was constructed by a commercial cloning service (Synbio Technologies, USA). The *recA* coding sequence from *E. coli* MG1655 was inserted downstream of a T5 promoter in the low-copy plasmid backbone pUA66, which includes a strong mutated *lacI* repressor for tight regulation and a kanamycin resistance marker. The plasmid was verified by sequencing (Plasmidsaurus, USA) and transformed into MG1655 Δ*recA* cells for inducible expression studies.

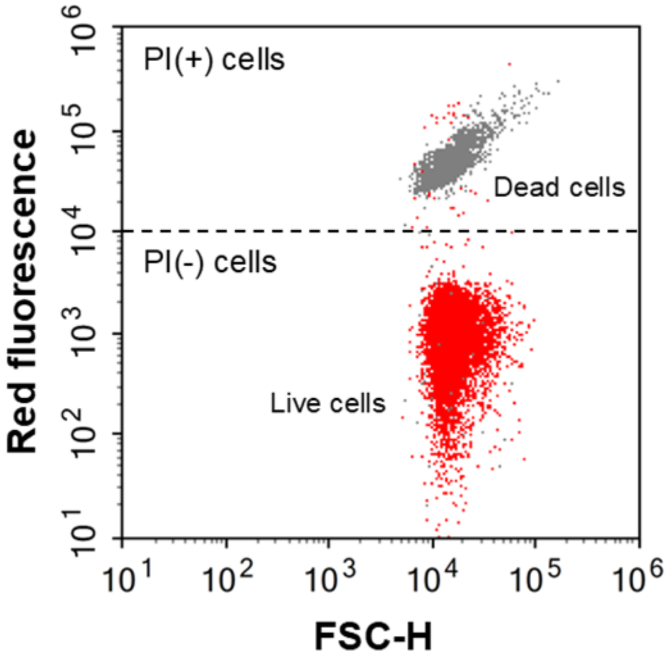

Fig. S8. PI staining of live and dead cells. Live cells (Red) and ethanol-treated dead cells (70% v/v, Grey) were stained with PI and analyzed by flow cytometry to assess live and dead cell populations. The figure shows a representative flow cytometry diagram. Similar results were observed across all independent biological replicates, n=4.

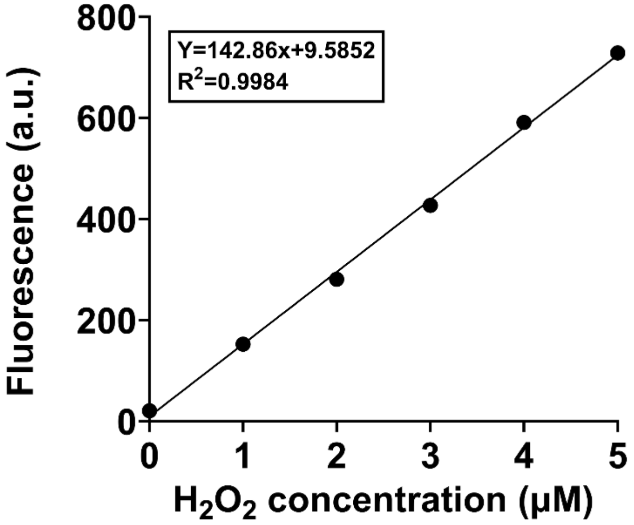

Fig. S9. Standard curve for H_2O_2 measurement using the Amplex Red Hydrogen Peroxide/Peroxidase Assay Kit. The standard curve was generated using a 20 mM Hydrogen Peroxide (H_2O_2) working solution, prepared by dissolving 3.0% (0.88 M) H_2O_2 in 1X Reaction Buffer. This solution was then diluted in 1X Reaction Buffer to achieve H_2O_2 concentrations ranging from 0 to 10 µM, in 50 µL aliquots. To each microplate well containing the standards, 50 µL of Amplex® Red reagent/HRP working solution was added. After a 30-minute incubation, fluorescence was measured using a microplate reader with excitation in the range of 530–560 nm and emission detection at ~590 nm.

Supplementary tables

Table S1: Promoter and single-deletion knockout libraries used in this study

Bacterial Strains	Source
E. coli K-12 MG1655 *pMSs201* P_{uvrA}-*gfp*	Promoter library from Horizon Discovery, Lafayette, CO, USA
E. coli K-12 MG1655 *pMSs201* P_{dps}-*gfp*	
E. coli K-12 MG1655 *pMSs201* P_{soxS}-*gfp*	
E. coli K-12 MG1655 *pMSs201* P_{recX}-*gfp*	
E. coli K-12 MG1655 *pMSs201* P_{molR}-*gfp*	
E. coli K-12 MG1655 *pMSs201* P_{polA}-*gfp*	
E. coli K-12 MG1655 *pMSs201* P_{dinG}-*gfp*	
E. coli K-12 MG1655 *pMSs201* P_{mutS}-*gfp*	
E. coli K-12 MG1655 *pMSs201* P_{mutH}-*gfp*	
E. coli K-12 MG1655 *pMSs201* P_{mutY}-*gfp*	
E. coli K-12 MG1655 *pMSs201* P_{ybfE}-*gfp*	
E. coli K-12 MG1655 *pMSs201* P_{sbmC}-*gfp*	
E. coli K-12 MG1655 *pMSs201* P_{mutM}-*gfp*	
E. coli K-12 MG1655 *pMSs201* P_{yjiW}-*gfp*	
E. coli K-12 MG1655 *pMSs201* P_{mutT}-*gfp*	
E. coli K-12 MG1655 *pMSs201* P_{dinB}-*gfp*	
E. coli K-12 MG1655 *pMSs201* P_{ftsK}-*gfp*	
E. coli K-12 MG1655 *pMSs201* P_{sodB}-*gfp*	
E. coli K-12 MG1655 *pMSs201* P_{recN}-*gfp*	
E. coli K-12 MG1655 *pMSs201* P_{rmuC}-*gfp*	
E. coli K-12 MG1655 *pMSs201* P_{dinJ}-*gfp*	
E. coli K-12 MG1655 *pMSs201* P_{yebG}-*gfp*	
E. coli K-12 MG1655 *pMSs201* P_{sulA}-*gfp*	
E. coli K-12 MG1655 *pMSs201* P_{soxR}-*gfp*	
E. coli K-12 MG1655 *pMSs201* P_{ahpC}-*gfp*	
E. coli K-12 MG1655 *pMSs201* P_{katE}-*gfp*	
E. coli K-12 MG1655 *pMSs201* P_{sodC}-*gfp*	
E. coli K-12 MG1655 *pMSs201* P_{ahpF}-*gfp*	
E. coli K-12 MG1655 *pMSs201* P_{yhiL}-*gfp*	
E. coli K-12 MG1655 *pMSs201* P_{uvrC}-*gfp*	
E. coli K-12 MG1655 *pMSs201* P_{oxyR}-*gfp*	
E. coli K-12 MG1655 *pMSs201* P_{polB}-*gfp*	
E. coli K-12 MG1655 *pMSs201* P_{uvrD}-*gfp*	
E. coli K-12 MG1655 *pMSs201* P_{sodA}-*gfp*	
E. coli K-12 MG1655 *pMSs201* P_{lexA}-*gfp*	

E. coli K-12 MG1655 *pMSs201* P_{recA}-*gfp*	
E. coli K-12 MG1655 *pMSs201* P_{lon}-*gfp*	
E. coli K-12 MG1655 *pMSs201* P_{umuD}-*gfp*	
E. coli K-12 MG1655 *pMSs201* P_{ssb}-*gfp*	
E. coli K-12 BW25113 Δ*fnr*	
E. coli K-12 BW25113 Δ*soxR*	
E. coli K-12 BW25113 Δ*soxS*	
E. coli K-12 BW25113 Δ*umuD*	
E. coli K-12 BW25113 Δ*recA*	
E. coli K-12 BW25113 Δ*ruvB*	
E. coli K-12 BW25113 Δ*uvrD*	
E. coli K-12 BW25113 Δ*sodB*	
E. coli K-12 BW25113 Δ*sodC*	
E. coli K-12 BW25113 Δ*ahpF*	
E. coli K-12 BW25113 Δ*katG*	
E. coli K-12 BW25113 Δ*sodA*	
E. coli K-12 BW25113 Δ*ygjF*	
E. coli K-12 BW25113 Δ*polA*	
E. coli K-12 BW25113 Δ*ruvA*	
E. coli K-12 BW25113 Δ*ruvC*	
E. coli K-12 BW25113 Δ*umuC*	
E. coli K-12 BW25113 Δ*uvrA*	Keio collection, Cat# OEC4987
E. coli K-12 BW25113 Δ*alkA*	
E. coli K-12 BW25113 Δ*ahpC*	
E. coli K-12 BW25113 Δ*sulA*	
E. coli K-12 BW25113 Δ*katE*	
E. coli K-12 BW25113 Δ*uvrB*	
E. coli K-12 BW25113 Δ*nei*	
E. coli K-12 BW25113 Δ*dinB*	
E. coli K-12 BW25113 Δ*recB*	
E. coli K-12 BW25113 Δ*nth*	
E. coli K-12 BW25113 Δ*uvrC*	
E. coli K-12 BW25113 Δ*fur*	
E. coli K-12 BW25113 Δ*ligB*	
E. coli K-12 BW25113 Δ*ung*	
E. coli K-12 BW25113 Δ*tag*	
E. coli K-12 BW25113 Δ*recN*	
E. coli K-12 BW25113 Δ*mutY*	
E. coli K-12 BW25113 Δ*ycaQ*	

E. coli K-12 BW25113 Δ*mutM*	
E. coli K-12 BW25113 Δ*recC*	
E. coli K-12 BW25113 Δ*polB*	
E. coli K-12 BW25113 Δ*recD*	
E. coli K-12 BW25113 Δ*oxyR*	
E. coli K-12 BW25113 Δ*mutS*	
E. coli K-12 BW25113 Δ*mutH*	
E. coli K-12 BW25113 Δ*mutL*	
E. coli K-12 BW25113 Δ*exoI*	
E. coli K-12 BW25113 Δ*phrB*	
E. coli K-12 BW25113 Δ*lacI*	

Table S2: Bacterial plasmids and strains used in this study

Bacterial Strains	Source
E. coli K-12 MG1655 pUA66 P*recA-gfp*	Gift from Dr. Mark P. Brynildsen
E. coli K-12 MG1655 pUA66-*gfp*	Gift from Dr. Mark P. Brynildsen
E. coli K-12 MG1655 Wild type	Gift from Dr. Mark P. Brynildsen
E. coli K-12 MG1655 Δ*sulA*	Previous study
E. coli K-12 MG1655 Δ*tisB*	Previous study
E. coli K-12 MG1655 Δ*recA*	Previous study
E. coli K-12 MG1655 Δ*sulA*Δ*tisB*	This study
E. coli K-12 MG1655 Δ*recN*	This study
E. coli K-12 MG1655 Δ*rmuC*	This study
E. coli K-12 MG1655 Δ*polB*	This study
E. coli K-12 MG1655 Δ*dinB*	This study
E. coli K-12 MG1655 Δ*sbmC*	This study
E. coli K-12 MG1655 Δ*ybfE*	This study
E. coli K-12 MG1655 Δ*polB*Δ*dinB*	This study
E. coli K-12 MG1655 Δ*recN*Δ*rmuC*	This study
E. coli K-12 MG1655 Δ*recN*Δ*polB*	This study
E. coli K-12 MG1655 Δ*recN*Δ*dinB*	This study
E. coli K-12 MG1655 Δ*recN*Δ*rmuC*Δ*polB*	This study
E. coli K-12 MG1655 Δ*recN*Δ*rmuC*Δ*dinB*	This study
E. coli K-12 MG1655 Δ*recB*	This study
E. coli K-12 MG1655 Δ*ruvC*	This study
E. coli K-12 MG1655 Δ*umuC*	This study
E. coli K-12 MG1655 Δ*umuD*	This study

E. coli K-12 MG1655 Δ*katE*	This study
E. coli K-12 MG1655 Δ*uvrA*	This study
E. coli K-12 MG1655 Δ*uvrD*	Previous study
E. coli K-12 MG1655 Δ*mutY*	This study
E. coli K-12 MG1655 Δ*recA*+pUA66-*recA*	This study
E. coli K-12 MG1655 Δ*recA*+ pUA66-E.V.	This study

Table S3: Oligonucleotides used to generate single and multi-deletion mutants

**Oligonucleotides to generate gene deletions**			
**Mutation**	**Forward Primer (5' to 3'):**	**Reverse Primer (5' to 3')**	**Source**
Δ*recN*::KAN^R	ACACAATAACAGTAATGG TTTTTCATACAGGAAAAC GACTGTGTAGGCTGGAGC TGCTTC	CGTTTTGCTGTTTACTC TGACCGTGAAGCAGGA AAAAAGTTTAACGGCT GACATGGGAAT	Integrated DNA Technologies, Inc.
Δ*rmuC*::KAN^R	CAGGAAATGCCTTTCCA ACTGGACGTTTGTACAG CACAATTCTATTTTGTG CGGGTAAGTTGTTGCGT CAGGAGGCGTTGTGTA GGCTGGAGCTGCTTC	TCAAAAAATTGTTCC AGAAGTGTAACAGAT TGGGCGTCGATGCCC TAGATTTCTACCCGG CTTAACTACTCCCAA TGGGTTAACGGCTGA CATGGGAAT	Integrated DNA Technologies, Inc.
Δ*polB*::KAN^R	CAGGCTATACTCAAGCC TGGTTTTTTGATGGATT TTCAGCGTGTAGGCTGG AGCTGCTTC	TCACGCATCAAAATG GTATCTGGCGAACTC TTTTTTTTGCTTAACG GCTGACATGGGAAT	Integrated DNA Technologies, Inc.
Δ*dinB*::KAN^R	ACGCGTTAAATGCTGA ATCTTTACGCATTTCTC AAACCCTGAAATCACT GTATACTTTA CCAGTGTTGAGAGGTG AGCA GTGTAGGCTGGAGCTG CTTC	CCGATTTTTCAGCGA GAATTCGATGCATAC AGTGATACCCTCATA ATAATGCACACCAGA ATATACATAATAGTA TACATTAACGGCTGA CATGGGAAT	Integrated DNA Technologies, Inc.
Δ*sbmC*::KAN^R	CAACTATACTGTATATA AAAACAGTATCAATGG AGGCGTCGTGTAGGCT GGAGCTGCTTC	AAAGAGTGGTCATCG CGTTAACACACCGCC CTGAGATGAATTAAC GGCTGACATGGGAA	Integrated DNA Technologies, Inc.
Δ*ybfE*::KAN^R	AGAGGCCTGGCTGATT GTTTCCCCCGAAGTCAC CAAGATCGTGTAGGCT GGAGCTGCTTC	ACGAGCAGGACTGCA CACTGTGCTACATGA AAGTGGAAATTTAAC GGCTGACATGGGAAT	Integrated DNA Technologies, Inc.

Δ*recB*::KAN^{R}	AGCGCGTTGCAGCAAACAATGCCCCTGATGAGTGAAAAGAGTGTAGGCTGGAGCTGCTTC	GCGGGCGTAGCTGTTTGTGCTCCACAGCTTCCAGTAATTGCTTTTGCAATTTCATTAACGGCTGACATGGGAAT	Integrated DNA Technologies, Inc.
Δ*ruvC*::KAN^{R}	CTCTGATGAGGCCTGCTAAACAGCAAAACGGAGACGCGTGGTGTAGGCTGGAGCTGCTTC	GTGGAAACGCCTCAGCCGGAACTGACCGAGGCGGTATAACTTAACGGCTGACATGGGAAT	Integrated DNA Technologies, Inc.
Δ*umuC*::KAN^{R}	TCTTTGGTGTGGTGATCCACGTCGTTAAGGCGATGCGCTGGTGTAGGCTGGAGCTGCTTC	TCGGCGCTCCTGCGGGAGCGCTTTTTCCTGCCGCTATATTTAACGGCTGACATGGGAA	Integrated DNA Technologies, Inc.
Δ*umuD*::KAN^{R}	GAACAGACTACTGTATATAAAAACAGTATAACTTCAGGCAGATTATTGTGTAGGCTGGAGCTGCTTC	CAGCTGGCATAAAACGCGTTTACATCACAGAGGGCAAACATTTAACGGCTGACATGGGAAT	Integrated DNA Technologies, Inc.
Δ*katE*::KAN^{R}	ACAGCGGCCCTTTCAGTAATAAATTAAGGAGACGAGTTCAGTGTAGGCTGGAGCTGCTTC	ATGTAAATCATTTGAGGCGGCGCAATTGCGCCGCCTCCCATTAACGGCTGACATGGGAA	Integrated DNA Technologies, Inc.
Δ*uvrA*::KAN^{R}	ATGCCACCGGGCAAAAAAGCGTTTAATCCGGGAAAGGTGAGTGTAGGCTGGAGCTGCTTC	CTCTGAAAGGAAAAGGCCGCTCAGAAAGCGGCCTTAACGATTAACGGCTGACATGGGAA	Integrated DNA Technologies, Inc.
Δ*mutY*::KAN^{R}	TGCTGCAATCTTGCCCCCAACAACAGTGAATTCGGTGACCGTGTAGGCTGGAGCTGCTTC	TCGTTCTGCTCATAAATCATCCTCTTTATCGACTCACGCGTTAACGGCTGACATGGGAAT	Integrated DNA Technologies, Inc.
Δ*phrB*::KAN^{R} (Trial 1)	CTTTGGCCGCGTGTGATTAACTTGCGCCATTCAGGAGTTTTGTGTAGGCTGGAGCTGCTTC	CAGACGCGTCAGGCAATCGAGCCCAGATGCCGGATGCGGCTTAACGGCTGACATGGGAAT	Integrated DNA Technologies, Inc.
Δ*phrB*::KAN^{R} (Trial 2)	AGGGCAGCGTTATTTCGAACTTTGGCCGCGTGTGATTAACTTGCGCCATTCAGGAGTTTTGTGTAGGCTGGAGCTGCTTC	CTGTTCGGTCGCTAATCCATTCGGCGCTCCTGCGGGAGCGCTTTTTTCCTGCCGCTATATTTAACGGCTGACATGGGAAT	Integrated DNA Technologies, Inc.

Oligonucleotides to verify gene deletions

**Mutation**	**External Forward Primer (5' to 3')**	**External Reverse Primer (5' to 3')**	**Internal Forward Primer (5' to 3')**	**Internal Reverse Primer (5' to 3')**	**Source**

Δ*recN*::KAN^R	GATTCGTCGCTGTGATTACCATC	GCTCTTCGTCCAGATCATCCT	GCACAACTGACCATCAGCAA	CAGTTGCTGCTGTTCTTCCAGTAG	Integrated DNA Technologies, Inc.
Δ*rmuC*::KAN^R	AAAGCCATGCGGTGAAAATC	GCTCTTCGTCCAGATCATCCT	GTTATTGCGTTGGTGGGTGT	GGGTTCAACGGGAATAAACA	Integrated DNA Technologies, Inc.
Δ*polB*::KAN^R	GTCAGTTAGCGCCGCAGTTA	GCTCTTCGTCCAGATCATCCT	GCGCAGGCAGGTTTTATCTT	GGTTTATCTTCGGCGAAACG	Integrated DNA Technologies, Inc.
Δ*dinB*::KAN^R	GACCAAAAGTGCGTCCGATA	GCTCTTCGTCCAGATCATCCT	GTGGATATGGACTGCTTTTTCG	GGCGTTCATCCCAGGTTTTA	Integrated DNA Technologies, Inc.
Δ*sbmC*::KAN^R	GGTTACCCTGGAATCTGTCG	GCTCTTCGTCCAGATCATCCT	GAAGAGAAACGTACCGTTGCAG	GCTGCACCGCAACATACATT	Integrated DNA Technologies, Inc.
Δ*ybfE*::KAN^R	TACGCGCTATCCGTCGCTAT	GCTCTTCGTCCAGATCATCCT	AACAAACGGACCGTACGACA	GCCAGTTGCTGCATTAACATCT	Integrated DNA Technologies, Inc.
Δ*recB*::KAN^R	TTCTTCCATCAGGCGGTGGT	GCTCTTCGTCCAGATCATCCT	ATCCACTGTACGAACGCCTG	AAATTCGGTACTGCTGGGGG	Integrated DNA Technologies, Inc.
Δ*ruvC*::KAN^R	GAAACCGCACCGAAACTGAT	GCTCTTCGTCCAGATCATCCT	GGCTATTATTCTCGGCATTGATCC	AACGTGGCAGTGGGTGATAG	Integrated DNA Technologies, Inc.
Δ*umuC*::KAN^R	CTGTTGACGGCGAGTTTACG	GCTCTTCGTCCAGATCATCCT	AGCTGTGAGACGGTGTTTCG	GATCGCGTAGCAGCGTTAAT	Integrated DNA Technologies, Inc.
Δ*umuD*::KAN^R	TTTTCATTTCTGCTGGATGAGC	GCTCTTCGTCCAGATCATCCT	TCTTGTTCAGTGTGGCTTTCC	TGGATCACCACACCAAAGACA	Integrated DNA Technologies, Inc.
Δ*katE*::KAN^R	GTTTAGCCGATTTAGCCCCTG	GCTCTTCGTCCAGATCATCCT	ATCAGCCGCTCACGGTTATT	TATGGTAAGGGCAGGTCGGA	Integrated DNA Technologies, Inc.

Δ*uvrA*::KAN^R	F_Ex_sodA: CACAACA CTCCGGGT AATGC	GCTCTTC GTCCAGA TCATCCT	TGAATCCC TTTCCGCC TACG	ACGGATG ACGACGA ATGGAG	Integrated DNA Technologies, Inc.
Δ*mutY*::KAN^R	CAAATTCC GGTGAAA TGACG	GCTCTTC GTCCAGA TCATCCT	GCCCAGGT TCTGGACT GGTA	CGACACG GGAAGCC ACATAG	Integrated DNA Technologies, Inc.
Δ*phrB*::KAN^R (Trial 1)	TCCTGACG CCTGGCTT TCAG	GCTCTTC GTCCAGA TCATCCT	ATATCGCT ACACCACG CCAG	AACAAGC GATGCAA GCACTG	Integrated DNA Technologies, Inc.
Δ*phrB*::KAN^R (Trial 2)	TTCCACAG AACGCCA GCAGC	GCTCTTC GTCCAGA TCATCCT	CCAGTGGG CGACGCAT AACA	AGCGGCA TCAACAA TCGGGT	Integrated DNA Technologies, Inc.
