# Supplementary material for: UV-Induced DNA Repair Mechanisms and Their Effects on Mutagenesis and Culturability in Escherichia coli
Source: bioRxiv. 2024 Nov 14:2024.11.14.623584. Preprint. [Version 1] doi: 10.1101/2024.11.14.623584 (PMC11601333; doi:10.1101/2024.11.14.623584)
Supplement: Supplementary file 1 [file NIHPP2024.11.14.623584v1-supplement-1.pdf]

bioRxiv preprint doi: https://doi.org/10.1101/2024.11.14.623584; this version posted November 14, 2024. The copyright holder for this preprint (which was not certified by peer review) is the author/funder, who has granted bioRxiv a license to display the preprint in perpetuity. It is made available under aCC-BY 4.0 International license.

Supplementary Information

UV-Induced DNA Repair Mechanisms and Their Effects on Mutagenesis and Culturability in *Escherichia coli*

Sreyashi Ghosh[1], and Mehmet A. Orman[1]*

[1]William A. Brookshire Department of Chemical and Biomolecular Engineering, University of Houston, Houston, TX, USA

*Correspondence to: S222 Engineering Bldg 1, 4226 Martin Luther King Boulevard, Houston, TX 77204-4004, Phone: 713-743-6785, Email: morman@central.uh.edu

Supplementary figures

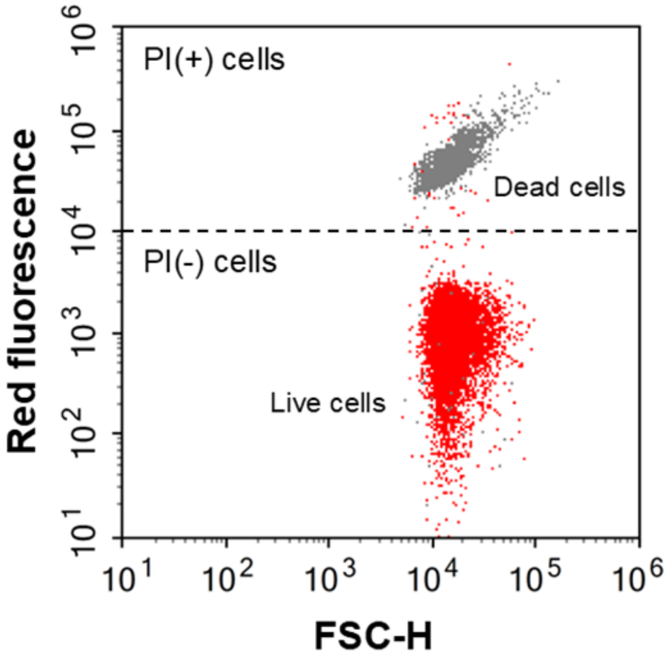

Fig. S1. PI staining of live and dead cells. Live cells (Red) and ethanol-treated dead cells (70% v/v, Grey) were stained with PI and analyzed by flow cytometry to assess live and dead cell populations. The figure shows a representative flow cytometry diagram. Similar results were observed across all independent biological replicates, n=4.

bioRxiv preprint doi: https://doi.org/10.1101/2024.11.14.623584; this version posted November 14, 2024. The copyright holder for this preprint (which was not certified by peer review) is the author/funder, who has granted bioRxiv a license to display the preprint in perpetuity. It is made available under aCC-BY 4.0 International license.

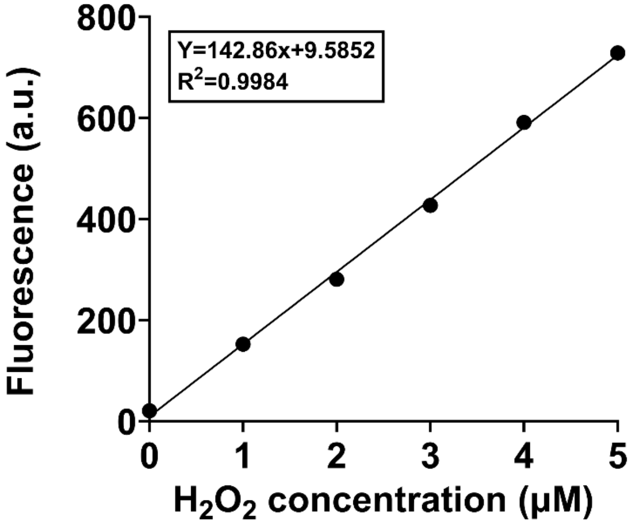

Fig. S2. Standard curve for H_2O_2 measurement using the Amplex Red Hydrogen Peroxide/Peroxidase Assay Kit. The standard curve was generated using a 20 mM Hydrogen Peroxide (H_2O_2) working solution, prepared by dissolving 3.0% (0.88 M) H_2O_2 in 1X Reaction Buffer. This solution was then diluted in 1X Reaction Buffer to achieve H_2O_2 concentrations ranging from 0 to 10 µM, in 50 µL aliquots. To each microplate well containing the standards, 50 µL of Amplex® Red reagent/HRP working solution was added. After a 30-minute incubation, fluorescence was measured using a microplate reader with excitation in the range of 530–560 nm and emission detection at ~590 nm.

bioRxiv preprint doi: https://doi.org/10.1101/2024.11.14.623584; this version posted November 14, 2024. The copyright holder for this preprint (which was not certified by peer review) is the author/funder, who has granted bioRxiv a license to display the preprint in perpetuity. It is made available under aCC-BY 4.0 International license.

Supplementary tables

Table S1: Promoter library and single-deletion knockout library used in this study.

Bacterial Strains	Source
E. coli K-12 MG1655 *pMSs201* P_{uvrA}-*gfp*	Promoter library from Horizon Discovery, Lafayette, CO, USA
E. coli K-12 MG1655 *pMSs201* P_{dps}-*gfp*	
E. coli K-12 MG1655 *pMSs201* P_{soxS}-*gfp*	
E. coli K-12 MG1655 *pMSs201* P_{recX}-*gfp*	
E. coli K-12 MG1655 *pMSs201* P_{molR}-*gfp*	
E. coli K-12 MG1655 *pMSs201* P_{polA}-*gfp*	
E. coli K-12 MG1655 *pMSs201* P_{dinG}-*gfp*	
E. coli K-12 MG1655 *pMSs201* P_{mutS}-*gfp*	
E. coli K-12 MG1655 *pMSs201* P_{mutH}-*gfp*	
E. coli K-12 MG1655 *pMSs201* P_{mutY}-*gfp*	
E. coli K-12 MG1655 *pMSs201* P_{ybfE}-*gfp*	
E. coli K-12 MG1655 *pMSs201* P_{sbmC}-*gfp*	
E. coli K-12 MG1655 *pMSs201* P_{mutM}-*gfp*	
E. coli K-12 MG1655 *pMSs201* P_{yjiW}-*gfp*	
E. coli K-12 MG1655 *pMSs201* P_{mutT}-*gfp*	
E. coli K-12 MG1655 *pMSs201* P_{dinB}-*gfp*	
E. coli K-12 MG1655 *pMSs201* P_{ftsK}-*gfp*	
E. coli K-12 MG1655 *pMSs201* P_{sodB}-*gfp*	
E. coli K-12 MG1655 *pMSs201* P_{recN}-*gfp*	
E. coli K-12 MG1655 *pMSs201* P_{rmuC}-*gfp*	
E. coli K-12 MG1655 *pMSs201* P_{dinJ}-*gfp*	
E. coli K-12 MG1655 *pMSs201* P_{yebG}-*gfp*	
E. coli K-12 MG1655 *pMSs201* P_{sulA}-*gfp*	
E. coli K-12 MG1655 *pMSs201* P_{soxR}-*gfp*	
E. coli K-12 MG1655 *pMSs201* P_{ahpC}-*gfp*	
E. coli K-12 MG1655 *pMSs201* P_{katE}-*gfp*	
E. coli K-12 MG1655 *pMSs201* P_{sodC}-*gfp*	
E. coli K-12 MG1655 *pMSs201* P_{ahpF}-*gfp*	
E. coli K-12 MG1655 *pMSs201* P_{yhiL}-*gfp*	
E. coli K-12 MG1655 *pMSs201* P_{uvrC}-*gfp*	
E. coli K-12 MG1655 *pMSs201* P_{oxyR}-*gfp*	
E. coli K-12 MG1655 *pMSs201* P_{polB}-*gfp*	
E. coli K-12 MG1655 *pMSs201* P_{uvrD}-*gfp*	
E. coli K-12 MG1655 *pMSs201* P_{sodA}-*gfp*	
E. coli K-12 MG1655 *pMSs201* P_{lexA}-*gfp*	
E. coli K-12 MG1655 *pMSs201* P_{recA}-*gfp*	

bioRxiv preprint doi: https://doi.org/10.1101/2024.11.14.623584; this version posted November 14, 2024. The copyright holder for this preprint (which was not certified by peer review) is the author/funder, who has granted bioRxiv a license to display the preprint in perpetuity. It is made available under aCC-BY 4.0 International license.

E. coli K-12 MG1655 *pMSs201* P_{lon}-*gfp*	
E. coli K-12 MG1655 *pMSs201* P_{umuD}-*gfp*	
E. coli K-12 MG1655 *pMSs201* P_{ssb}-*gfp*	
E. coli K-12 BW25113 Δ*fnr*	Keio collection, Cat# OEC4987
E. coli K-12 BW25113 Δ*soxR*	
E. coli K-12 BW25113 Δ*soxS*	
E. coli K-12 BW25113 Δ*umuD*	
E. coli K-12 BW25113 Δ*recA*	
E. coli K-12 BW25113 Δ*ruvB*	
E. coli K-12 BW25113 Δ*uvrD*	
E. coli K-12 BW25113 Δ*sodB*	
E. coli K-12 BW25113 Δ*sodC*	
E. coli K-12 BW25113 Δ*ahpF*	
E. coli K-12 BW25113 Δ*katG*	
E. coli K-12 BW25113 Δ*sodA*	
E. coli K-12 BW25113 Δ*ygjF*	
E. coli K-12 BW25113 Δ*polA*	
E. coli K-12 BW25113 Δ*ruvA*	
E. coli K-12 BW25113 Δ*ruvC*	
E. coli K-12 BW25113 Δ*umuC*	
E. coli K-12 BW25113 Δ*uvrA*	
E. coli K-12 BW25113 Δ*alkA*	
E. coli K-12 BW25113 Δ*ahpC*	
E. coli K-12 BW25113 Δ*sulA*	
E. coli K-12 BW25113 Δ*katE*	
E. coli K-12 BW25113 Δ*uvrB*	
E. coli K-12 BW25113 Δ*nei*	
E. coli K-12 BW25113 Δ*dinB*	
E. coli K-12 BW25113 Δ*recB*	
E. coli K-12 BW25113 Δ*nth*	
E. coli K-12 BW25113 Δ*uvrC*	
E. coli K-12 BW25113 Δ*fur*	
E. coli K-12 BW25113 Δ*ligB*	
E. coli K-12 BW25113 Δ*ung*	
E. coli K-12 BW25113 Δ*tag*	
E. coli K-12 BW25113 Δ*recN*	
E. coli K-12 BW25113 Δ*mutY*	
E. coli K-12 BW25113 Δ*ycaQ*	
E. coli K-12 BW25113 Δ*mutM*	

bioRxiv preprint doi: https://doi.org/10.1101/2024.11.14.623584; this version posted November 14, 2024. The copyright holder for this preprint (which was not certified by peer review) is the author/funder, who has granted bioRxiv a license to display the preprint in perpetuity. It is made available under aCC-BY 4.0 International license.

E. coli K-12 BW25113 Δ*recC*	
E. coli K-12 BW25113 Δ*polB*	
E. coli K-12 BW25113 Δ*recD*	
E. coli K-12 BW25113 Δ*oxyR*	
E. coli K-12 BW25113 Wild type	

Table S2: Bacterial plasmid and strains used in this study.

Bacterial Strains	Source
E. coli K-12 MG1655 pUA66 P*recA-gfp*	Gift from Dr. Mark P. Brynildsen
E. coli K-12 MG1655 pUA66-*gfp*	Gift from Dr. Mark P. Brynildsen
E. coli K-12 MG1655 Wild type	Gift from Dr. Mark P. Brynildsen
E. coli K-12 MG1655 Δ*sulA*	Previous study
E. coli K-12 MG1655 Δ*tisB*	Previous study
E. coli K-12 MG1655 Δ*recA*	Previous study
E. coli K-12 MG1655 Δ*sulA*Δ*tisB*	This study
E. coli K-12 MG1655 Δ*recN*	This study
E. coli K-12 MG1655 Δ*rmuC*	This study
E. coli K-12 MG1655 Δ*polB*	This study
E. coli K-12 MG1655 Δ*dinB*	This study
E. coli K-12 MG1655 Δ*sbmC*	This study
E. coli K-12 MG1655 Δ*ybfE*	This study
E. coli K-12 MG1655 Δ*polB*Δ*dinB*	This study
E. coli K-12 MG1655 Δ*recN*Δ*rmuC*	This study
E. coli K-12 MG1655 Δ*recN*Δ*polB*	This study
E. coli K-12 MG1655 Δ*recN*Δ*dinB*	This study
E. coli K-12 MG1655 Δ*recN*Δ*rmuC*Δ*polB*	This study
E. coli K-12 MG1655 Δ*recN*Δ*rmuC*Δ*dinB*	This study
E. coli K-12 MG1655 Δ*recB*	This study
E. coli K-12 MG1655 Δ*ruvC*	This study
E. coli K-12 MG1655 Δ*umuC*	This study
E. coli K-12 MG1655 Δ*umuD*	This study
E. coli K-12 MG1655 Δ*katE*	This study
E. coli K-12 MG1655 Δ*uvrA*	This study
E. coli K-12 MG1655 Δ*uvrD*	Previous study
E. coli K-12 MG1655 Δ*mutY*	This study

bioRxiv preprint doi: https://doi.org/10.1101/2024.11.14.623584; this version posted November 14, 2024. The copyright holder for this preprint (which was not certified by peer review) is the author/funder, who has granted bioRxiv a license to display the preprint in perpetuity. It is made available under aCC-BY 4.0 International license.

Table S3: Oligonucleotides used the generating single and multi-deletion mutants

**Oligonucleotides to generate gene deletions**			
**Mutation**	**Forward Primer (5' to 3'):**	**Reverse Primer (5' to 3')**	**Source**
Δ*recN*::KAN^{R}	ACACAATAACAGTAATGGTTTTTCATACAGGAAAACGACTGTGTAGGCTGGAGCTGCTTC	CGTTTTGCTGTTTACTCTGACCGTGAAGCAGGAAAAAAGTTTAACGGCTGACATGGGAAT	Integrated DNA Technologies, Inc.
Δ*rmuC*::KAN^{R}	CAGGAAATGCCTTTCCAACTGGACGTTTGTACAGCACAATTCTATTTGTGCGGGTAAGTTGTTGCGTCAGGAGGCGTTGTGTAGGCTGGAGCTGCTTC	TCAAAAAATTGTTCCAGAAGTGTAACAGATTGGGCGTCGATGCCCTAGATTTCTACCCGGCTTAACTACTCCCAATGGGTTAACGGCTGACATGGGAAT	Integrated DNA Technologies, Inc.
Δ*polB*::KAN^{R}	CAGGCTATACTCAAGCCTGGTTTTTTGATGGATTTTCAGCGTGTAGGCTGGAGCTGCTTC	TCACGCATCAAAATGGTATCTGGCGAACTCTTTTTTTTGCTTAACGGCTGACATGGGAAT	Integrated DNA Technologies, Inc.
Δ*dinB*::KAN^{R}	ACGCGTTAAATGCTGAATCTTTACGCATTTCTCAAACCCTGAAATCACTGTATACTTTACCAGTGTTGAGAGGTGAGCAGTGTAGGCTGGAGCTGCTTC	CCGATTTTTCAGCGAGAATTCGATGCATACAGTGATACCCTCATAATAATGCACACCAGAATATACATAATAGTATACATTAACGGCTGACATGGGAAT	Integrated DNA Technologies, Inc.
Δ*sbmC*::KAN^{R}	CAACTATACTGTATATAAAAACAGTATCAATGGAGGCGTCGTGTAGGCTGGAGCTGCTTC	AAAGAGTGGTCATCGCGTTAACACACCGCCCTGAGATGAATTAACGGCTGACATGGGAAT	Integrated DNA Technologies, Inc.
Δ*ybfE*::KAN^{R}	AGAGGCCTGGCTGATTGTTTCCCCCGAAGTCACCAAGATCGTGTAGGCTGGAGCTGCTTC	ACGAGCAGGACTGCACACTGTGCTACATGAAAGTGGAAATTTAACGGCTGACATGGGAAT	Integrated DNA Technologies, Inc.
Δ*recB*::KAN^{R}	AGCGCGTTGCAGCAAACAATGCCCCTGATGAGTGAAAAGAGTGTAGGCTGGAGCTGCTTC	GCGGGCGTAGCTGTTTGTGCTCCACAGCTTCCAGTAATTGCTTTTGCAATTTCATTAACGGCTGACATGGGAAT	Integrated DNA Technologies, Inc.
Δ*ruvC*::KAN^{R}	CTCTGATGAGGCCTGCTAAACAGCAAAACGGAGACGCGTGGTGTAGGCTGGAGCTGCTTC	GTGGAAACGCCTCAGCCGGAACTGACCGAGGCGGTATAACTTA	Integrated DNA Technologies, Inc.

bioRxiv preprint doi: https://doi.org/10.1101/2024.11.14.623584; this version posted November 14, 2024. The copyright holder for this preprint (which was not certified by peer review) is the author/funder, who has granted bioRxiv a license to display the preprint in perpetuity. It is made available under aCC-BY 4.0 International license.

		ACGGCTGACATGGGAAT	
Δ*umuC*::KAN^R	TCTTTGGTGTGGTGATCCACGTCGTTAAGGCGATGCGCTGGTGTAGGCTGGAGCTGCTTC	TCGGCGCTCCTGCGGGAGCGCTTTTTCCTGCCGCTATATTTAACGGCTGACATGGGAAT	Integrated DNA Technologies, Inc.
Δ*umuD*::KAN^R	GAACAGACTACTGTATATAAAAACAGTATAACTTCAGGCAGATTATTGTGTAGGCTGGAGCTGCTTC	CAGCTGGCATAAAACGCGTTTACATCACAGAGGGCAAACATTTAACGGCTGACATGGGAAT	Integrated DNA Technologies, Inc.
Δ*katE*::KAN^R	ACAGCGGCCCTTTCAGTAATAAATTAAGGAGACGAGTTCAGTGTAGGCTGGAGCTGCTTC	ATGTAAATCATTTGAGGCGGCGCAATTGCGCCGCCTCCCATTAACGGCTGACATGGGAAT	Integrated DNA Technologies, Inc.
Δ*uvrA*::KAN^R	ATGCCACCGGGCAAAAAAGCGTTTAATCCGGGAAAGGTGAGTGTAGGCTGGAGCTGCTTC	CTCTGAAAGGAAAAGGCCGCTCAGAAAGCGGCCTTAACGATTAACGGCTGACATGGGAAT	Integrated DNA Technologies, Inc.
Δ*mutY*::KAN^R	TGCTGCAATCTTGCCCCCAACAACAGTGAATTCGGTGACCGTGTAGGCTGGAGCTGCTTC	TCGTTCTGCTCATAAATCATCCTCTTTATCGACTCACGCGTTAACGGCTGACATGGGAAT	Integrated DNA Technologies, Inc.

Oligonucleotides to verify gene deletions

Mutation	External Forward Primer (5' to 3')	External Reverse Primer (5' to 3')	Internal Forward Primer (5' to 3')	Internal Reverse Primer (5' to 3')	Source
Δ*recN*::KAN^R	GATTCGTCGCTGTGATTACCATC	GCTCTTCGTCCAGATCATCCT	GCACAACTGACCATCAGCAA	CAGTTGCTGCTGTTCTTCCAGTAG	Integrated DNA Technologies, Inc.
Δ*rmuC*::KAN^R	AAAGCCATGCGGTGAAAATC	GCTCTTCGTCCAGATCATCCT	GTTATTGCGTTGGTGGGTGT	GGGTTCAACGGGAATAAACA	Integrated DNA Technologies, Inc.
Δ*polB*::KAN^R	GTCAGTTAGCGCCGCAGTTA	GCTCTTCGTCCAGATCATCCT	GCGCAGGCAGGTTTTATCTT	GGTTTATCTTCGGCGAAACG	Integrated DNA Technologies, Inc.
Δ*dinB*::KAN^R	GACCAAAAGTGCGTCCGATA	GCTCTTCGTCCAGATCATCCT	GTGGATATGGACTGCTTTTTCG	GGCGTTCATCCCAGGTTTTA	Integrated DNA Technologies, Inc.

bioRxiv preprint doi: https://doi.org/10.1101/2024.11.14.623584; this version posted November 14, 2024. The copyright holder for this preprint (which was not certified by peer review) is the author/funder, who has granted bioRxiv a license to display the preprint in perpetuity. It is made available under aCC-BY 4.0 International license.

Δ*sbmC*::KAN^{R}	GGTTACCCTGGAATCTGTCG	GCTCTTCGTCCAGATCATCCT	GAAGAGAAACGTACCGTTGCAG	GCTGCACCGCAACATACATT	Integrated DNA Technologies, Inc.
Δ*ybfE*::KAN^{R}	TACGCGCTATCCGTCGCTAT	GCTCTTCGTCCAGATCATCCT	AACAAACGGACCGTACGACA	GCCAGTTGCTGCATTAACATCT	Integrated DNA Technologies, Inc.
Δ*recB*::KAN^{R}	TTCTTCCATCAGGCGGTGGT	GCTCTTCGTCCAGATCATCCT	ATCCACTGTACGAACGCCTG	AAATTCGGTACTGCTGGGGG	Integrated DNA Technologies, Inc.
Δ*ruvC*::KAN^{R}	GAAACCGCACCGAAACTGAT	GCTCTTCGTCCAGATCATCCT	GGCTATTATTCTCGGCATTGATCC	AACGTGGCAGTGGGTGATAG	Integrated DNA Technologies, Inc.
Δ*umuC*::KAN^{R}	CTGTTGACGGCGAGTTTACG	GCTCTTCGTCCAGATCATCCT	AGCTGTGAGACGGTGTTTCG	GATCGCGTAGCAGCGTTAAT	Integrated DNA Technologies, Inc.
Δ*umuD*::KAN^{R}	TTTTCATTTCTGCTGGATGAGC	GCTCTTCGTCCAGATCATCCT	TCTTGTTCAGTGTGGCTTTCC	TGGATCACCACACCAAAGACA	Integrated DNA Technologies, Inc.
Δ*katE*::KAN^{R}	GTTTAGCCGATTTAGCCCCTG	GCTCTTCGTCCAGATCATCCT	ATCAGCCGCTCACGGTTATT	TATGGTAAGGGCAGGTCGGA	Integrated DNA Technologies, Inc.
Δ*uvrA*::KAN^{R}	F_Ex_sodA: CACAACACTCCGGGTAATGC	GCTCTTCGTCCAGATCATCCT	TGAATCCCTTTCCGCCTACG	ACGGATGACGACGAATGGAG	Integrated DNA Technologies, Inc.
Δ*mutY*::KAN^{R}	CAAATTCCGGTGAAATGACG	GCTCTTCGTCCAGATCATCCT	GCCCAGGTTCTGGACTGGTA	CGACACGGGAAGCCACATAG	Integrated DNA Technologies, Inc.
